# Site-Specific Acetylation of the Transcription Factor Protein Max Modulates Its DNA Binding Activity

**DOI:** 10.1021/acscentsci.4c00686

**Published:** 2024-06-12

**Authors:** Raj V. Nithun, Yumi Minyi Yao, Omer Harel, Shaimaa Habiballah, Ariel Afek, Muhammad Jbara

**Affiliations:** † School of Chemistry, Raymond and Beverly Sackler Faculty of Exact Sciences, Tel Aviv University, Tel Aviv, 69978 Israel; ‡ Department of Chemical and Structural Biology, Weizmann Institute of Science, Rehovot, 7610001, Israel

## Abstract

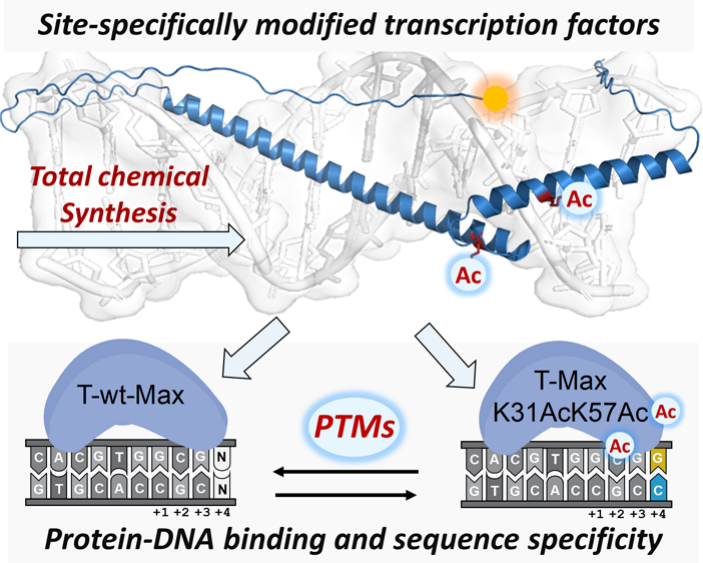

Chemical protein synthesis provides a powerful means to prepare novel modified proteins with precision down to the atomic level, enabling an unprecedented opportunity to understand fundamental biological processes. Of particular interest is the process of gene expression, orchestrated through the interactions between transcription factors (TFs) and DNA. Here, we combined chemical protein synthesis and high-throughput screening technology to decipher the role of post-translational modifications (PTMs), e.g., Lys-acetylation on the DNA binding activity of Max TF. We synthesized a focused library of singly, doubly, and triply modified Max variants including site-specifically acetylated and fluorescently tagged analogs. The resulting synthetic analogs were employed to decipher the molecular role of Lys-acetylation on the DNA binding activity and sequence specificity of Max. We provide evidence that the acetylation sites at Lys-31 and Lys-57 significantly inhibit the DNA binding activity of Max. Furthermore, by utilizing high-throughput binding measurements, we assessed the binding activities of the modified Max variants across diverse DNA sequences. Our results indicate that acetylation marks can alter the binding specificities of Max toward certain sequences flanking its consensus binding sites. Our work provides insight into the hidden molecular code of PTM-TFs and DNA interactions, paving the way to interpret gene expression regulation programs.

## Abstract 2

A focused library of site-specifically acetylated analogs of the transcription factor Max was chemically synthesized to dissect the molecular role of Lys-acetylation in the DNA binding activity of Max.

## Introduction

**Figure 1:**
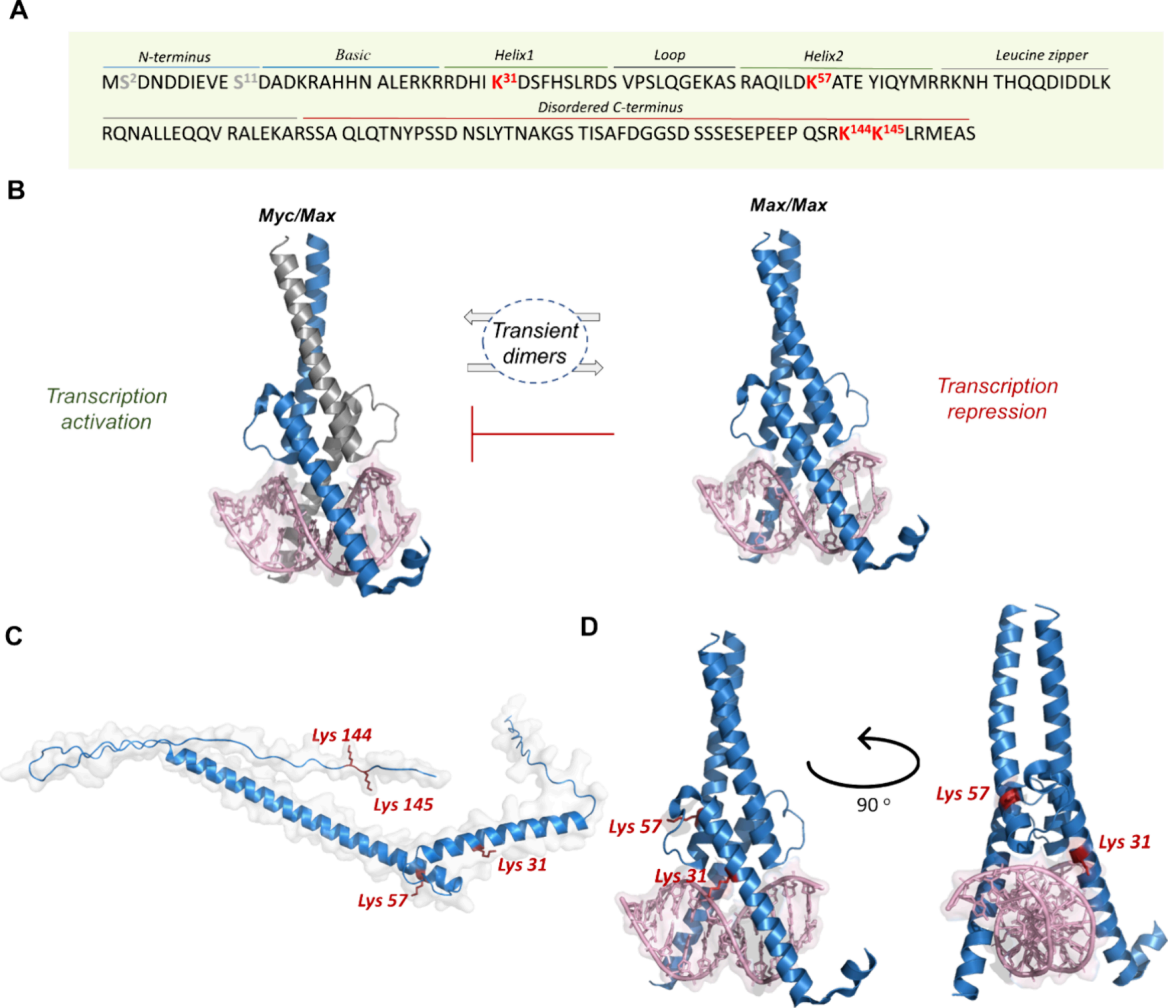
Max activity is regulated by post-translational modifications. (A) Max sequence (p21 isoform); the phosphorylation and acetylation sites are colored gray and red, respectively. (B) Max homodimerization or heterodimerization with Myc preferentially binds to the E-box DNA promoter to either activate or suppress gene transcription. (C) Predicted structure of full Max by alphafold (ID: P61244); acetylated sites are highlighted in red. (D) Max dimerization and binding to the E-box DNA; the acetylation sites at Lys 31 and Lys 57 are highlighted in red (PDB: 1HLO).

The basic helix–loop–helix (bHLH) transcription factors (TFs) constitute a large family of proteins found in all eukaryotes.^1,2^ The human bHLH TF family consists of more than 100 members that can assemble to form different types of oligomers that can bind to specific genes and control gene transcription.^3,4^ For example, to be active, the proto-oncogenic bHLH-TF Myc interacts with another bHLH-TF called Max through the leucine zipper motif (Figure 1).^5^ This heterodimerization enables the recognition of a core DNA motif called the enhancer box (E-box, 5′-CACGTG-3′) and initiates downstream gene transcription events.^6^ This complex regulates the expression of nearly 15% of the entire genome, and its overexpression can trigger tumorigenesis by uncontrolled cell growth, survival, and proliferation.^7^ Importantly, Max plays a critical role in regulating this process by forming transcriptionally active and inactive bHLH-TF complexes by “partner selection” mechanisms (Figure 1A,B).^8^ In addition to its essential heterodimerization with Myc, Max can homodimerize to form a transcriptionally inactive complex that competes for the same E-box DNA site, thereby playing an essential role in controlling gene expression.^9^

TFs are known to undergo dynamic post-translational modifications (PTMs) that can alter their folding, stability, cellular localization, and overall function.^10−12^ Importantly, Myc and Max TFs contain a basic domain and a leucine zipper motif that have been reported to undergo PTMs, such as phosphorylation and acetylation at multiple residues.^13−15^ These domains are known to mediate oligomerization and DNA binding activity. Moreover, the modification of these domains has been suggested to play an essential physiological role in regulating the DNA binding activity and gene transcription programs.^16^ In this regard, Max has been found to undergo an array of PTMs across multiple sites and various combinations, which can modulate its structure and function (Figure 1A).^13−15^ For example, Max phosphorylation of Ser 2 and Ser 11 via CK2 has been found to modulate its DNA binding activity.^13,14^ Another important PTM observed in Max is Lys acetylation. Max has been reported to undergo acetylation at several Lys residues (Lys 31, Lys 57, Lys 144, and Lys 145) in vitro via p300 histone acetyltransferase (HAT) (Figure 1C).^15^ It has been suggested that Lys acetylation plays an important role in controlling Max function by potentially regulating its cellular localization and DNA binding activity. However, the evidence supporting the impact of Max acetylation has been mostly indirect, investigated by monitoring the effect of the inhibition of histone deacetylases (HDACs) and by overexpression of the p300 coactivator/HAT or through mutagenesis (acetyl-Lys to Gln mutation). These approaches could result in indirect effects on the Max modification pattern, integrity, and function. The molecular role of the Lys acetylation pattern in the DNA binding and sequence specificity of Max, which is critical for gene expression regulation, remains unclear. A barrier to gaining molecular insights into the impact of the acetylation mark, especially on strategic Lys residues proximal to the DNA binding domain, is the difficulty in obtaining homogeneously acetylated and/or site-specifically labeled TF analogs at desired sites via common biological means.

Synthetic methods allow effective protein production with selective incorporation of desired modification at designated sites.^17−19^ The solid-phase peptide synthesis (SPPS) approach allows the desired PTM to be incorporated during the peptide elongation on a solid support, and the resulting synthetic polypeptide can be assembled in solution using chemoselective ligation approaches to provide full-length, homogeneously modified proteins.^20−23^ Synthetic and semisynthetic strategies have been successfully employed in the past decade to prepare numerous modified proteins for various applications.^24−28^ Some examples include phosphorylated, methylated, glycosylated, sulfonated, and ubiquitinated proteins.^29−33^ The ability to produce native proteins while preserving their functional integrity enabled fundamental biochemical and biophysical studies, which would otherwise be challenging to attempt.^34−37^ Here we report the chemical synthesis of homogeneously acetylated Max TF analogs via native chemical ligation (NCL) coupled with a desulfurization approach.^38,39^ To this end, we prepared a focused library of homogeneously modified Max TF variants including acetylated and fluorescently tagged analogs. The resulting analogs were employed to decode the impact of the acetylation mark in the leucine zipper and the basic domains on the DNA binding activity of Max. We found that Lys acetylation significantly inhibits the DNA binding activity of Max and reshapes the DNA sequence specificity in the flanking region of its consensus binding sites.

## Results and Discussion

### Chemical Synthesis of Site-Specifically Acetylated Max Variants

**Figure 2:**
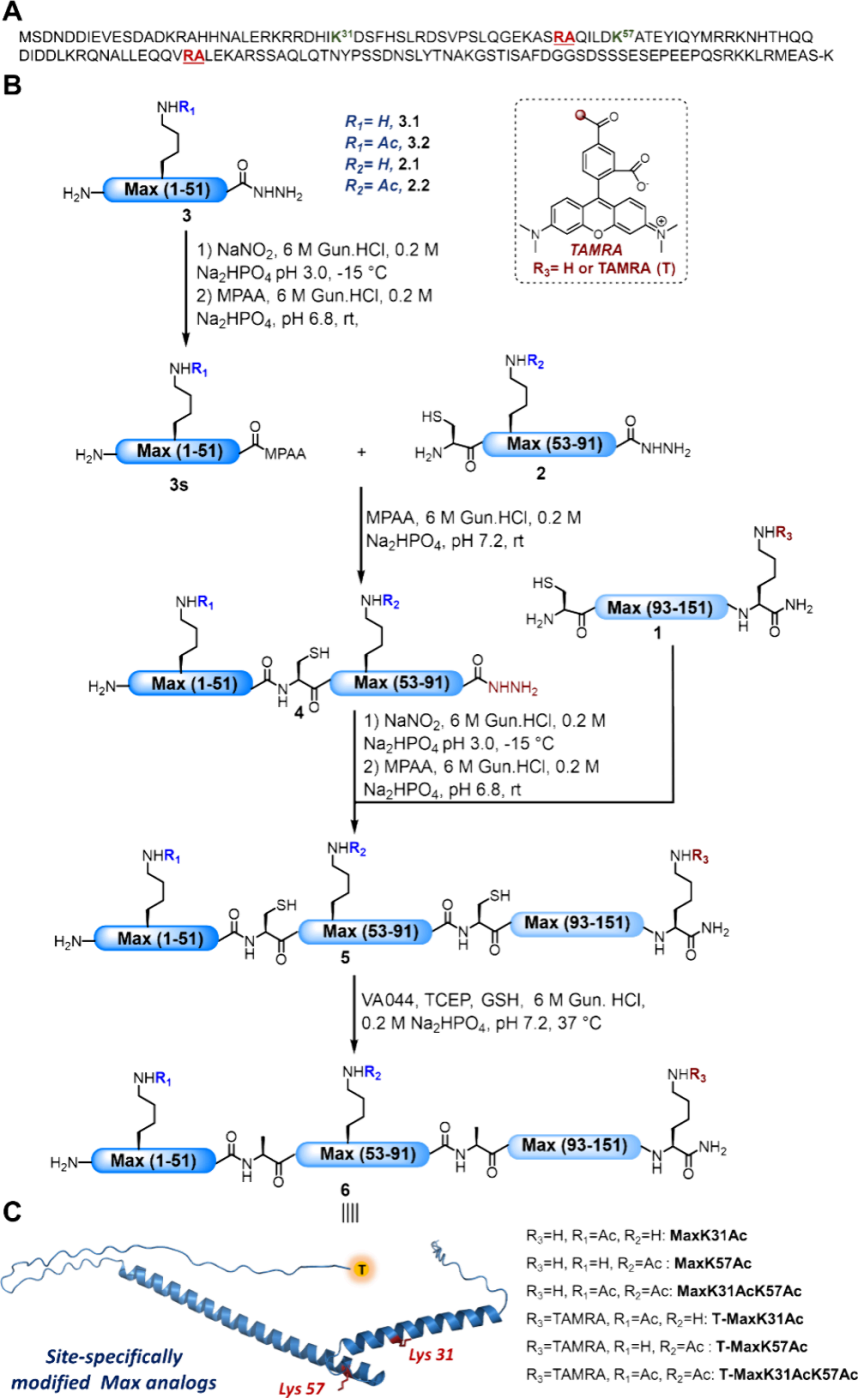
Chemical synthesis of site-specifically acetylated Max analogs. (A) Max sequence (p21 isoform); the ligation sites (Arg-Ala) are highlighted in red. The Met residues at positions 1, 65, and 148 were replaced by the homologous norleucine (Nle) residue to avoid Met oxidation. (B) Max synthesis through N-to-C-terminus ligation via native chemical ligation. (C) Modified Max analogs prepared in this study; predicted structure of Max by alphafold (ID: P61244).

To explore the impact of the acetylation mark, we decided to synthesize singly and doubly acetylated Max analogs that can potentially interfere with the DNA binding activity, e.g., Lys 31 and Lys 57, located in the leucine zipper and basic domains. To this end, we divided Max’s sequence into three segments and implemented a sequential N-to-C terminal ligation strategy at the Ala 52 and Ala 92 junctions (Figure 2).^40^ We prepared all peptide segments using standard Fmoc-SPPS and selectively incorporated the desired acetyl-Lys amino acid at positions Lys 31 and Lys 57 (see the SI, Section 4). The N-terminal Ala residues at the ligation sites (Ala 52 and Ala 92) were temporarily mutated to Cys to enable NCL at these sites, and the peptide thioesters were prepared through the peptide-hydrazide strategy (see the SI, Section 4).^41^ This design enabled the production of all segments: Cys-Max(93–151) segment **1**, Cys-Max(53–91)-NHNH_2_ segment **2.1**, Cys-MaxK57Ac(53–91)-NHNH_2_ segment **2.2**, Max(1–51)-NHNH_2_ segment **3.1**, and MaxK31Ac(1–51)-NHNH_2_ segment **3.2**. In addition, segment **1** was also prepared with a C-terminal fluorescent tag segment **1.1** as a reporter for in vitro high-throughput screening. We isolated the desired segments **1**, **1.1**, **2.1**, **2.2**, **3.1**, and **3.2** in 12%, 9%, 19%, 18%, 17%, and 15% yields, respectively, after RP-HPLC purification (see the SI, Section 4).

With all segments in hand, we initially carried out the synthesis of the doubly acetylated Max variant (**MaxK31AcK57Ac**). First, we converted segment **3.2**, MaxK31Ac(1–51)-NHNH_2_, to acyl-azide using NaNO_2_ in a 6 M Gun·HCl, 0.2 M Na_2_HPO_4_ buffer, pH 3.0, at −15 °C,^42^ and then treated the crude reaction mixture with 4-mercaptophenylacetic acid (MPAA) for the in situ thioesterification step (Figure 2).^38,43^ The thioester intermediate of **3.2** was then reacted with segment **2.2** (Cys-MaxK57Ac(53–91)-NHNH_2_) at room temperature for 1.5 h, followed by the addition of the reducing reagent tris(2-carboxyethyl)phosphine hydrochloride (TCEP·HCl). The progress of the ligation reaction was analyzed via HPLC-MS analysis, which was completed after 2 h to afford the desired ligated product **4** in 68% isolated yield after RP-HPLC purification (see the SI, Section 5). Next, we converted intermediate **4** to peptide thioester using NaNO_2_ and MPAA for the subsequent NCL reaction with segment **1** (Cys-Max(93–151)), which afforded the desired full-length protein **5** after 2 h. Then, we desalted the crude reaction to enable a one-pot desulfurization reaction in the presence of the radical initiator 2,2′-azobis[2-(2-imidazolin-2-yl)propane]dihydrochloride (VA044), TCEP, and glutathione (GSH) to convert Cys 52 and Cys 92 at ligation junctions to native Ala (see the SI, Section 5).^44,45^ We obtained the desired doubly modified full-length **MaxK31AcK57Ac** in 37% isolated yield for both synthetic steps.

**Figure 3:**
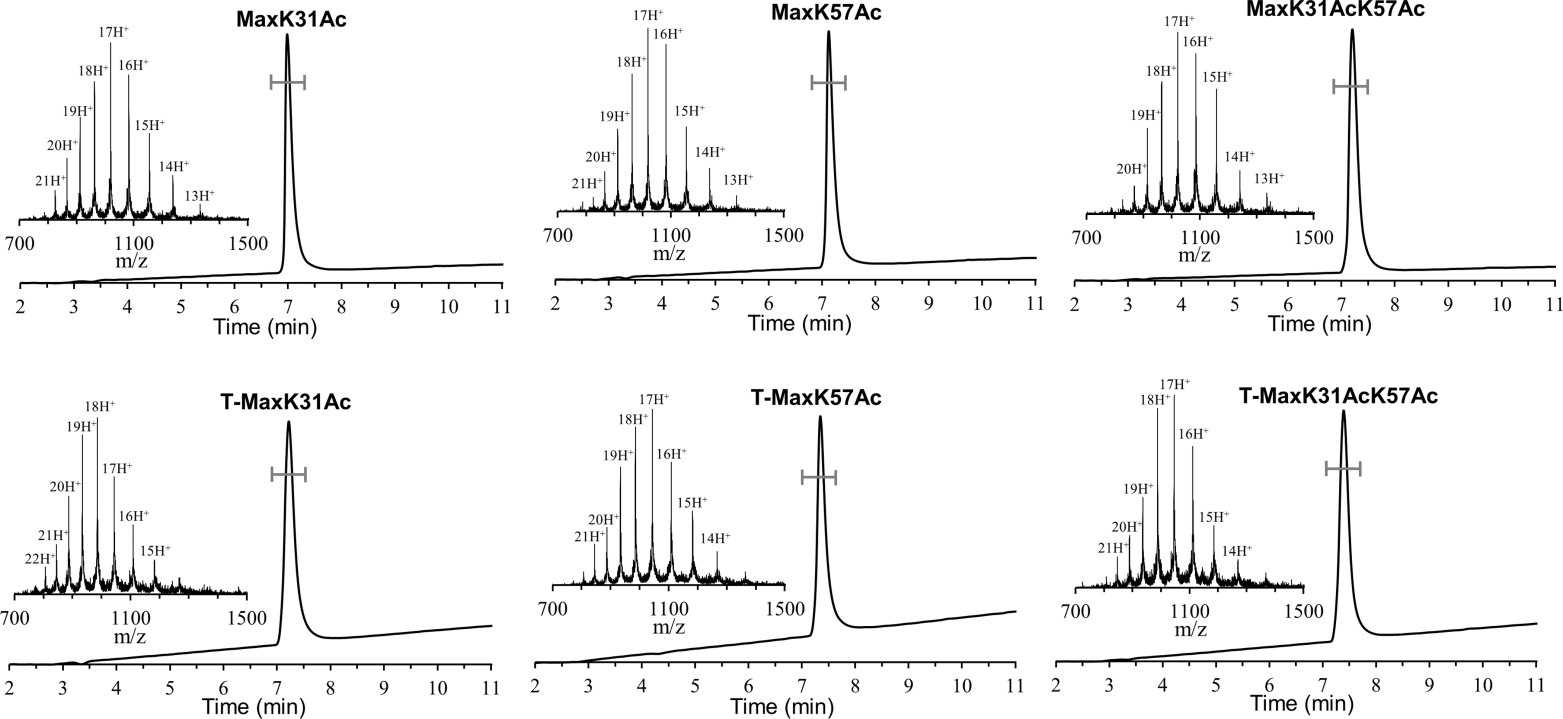
Analytical HPLC and mass traces of isolated Max analogs. The analytical RP-HPLC chromatograms and mass-to-charge (*m*/*z*) spectra of the isolated final products are depicted. **MaxK31Ac** with the observed mass 17313.1 ± 0.5 Da, calcd 17316.8 Da (average isotopes); **MaxK57Ac** with the observed mass 17313.7 ± 0.6 Da, calcd 17316.8 Da (average isotopes); **MaxK31K57Ac** with the observed mass 17354.8 ± 0.6 Da, calcd 17358.8 Da (average isotopes); **T-MaxK31Ac** with the observed mass 17724.6 ± 1.6 Da, calcd 17729.2 (average isotopes); **T-MaxK57Ac** with the observed mass 17725.2 ± 1.1 Da, calcd 17729.2 Da (average isotopes); **T-MaxK31K57Ac** with the observed mass 17767.8 ± 0.5 Da, calcd 17771.2 Da (average isotopes); data acquired over the marked region in the LC spectrum. The MS data are reported as *m*/*z* ratios. The experimental mass of each Max polypeptide chain variant was calculated from the *m*/*z* value of the individual multiply protonated charge states of the synthetic polypeptide chain variants.

Next, we employed the same synthetic design to prepare both singly acetylated Max analogs (**MaxK31Ac** and **MaxK57Ac**). First, we reacted segments **3.1** (Max(1–51)-NHNH_2_) and **3.2** (MaxK31Ac(1–51)-NHNH_2_) separately with segment **2.2** (Cys-MaxK57Ac(53–91)-NHNH_2_) and segment **2.1** (Cys-Max(53–91)-NHNH_2_) under the hydrazide-based NCL strategy using NaNO_2_ to afford the monoacetylated intermediates MaxK57Ac(1–91)-NHNH_2_ and MaxK31Ac(1–91)-NHNH_2_ in 73% and 55% isolated yields, respectively (see the SI, Section 5). Next, we ligated both intermediates with segment **1** separately to yield the desired full-length products after 2 h. Finally, both ligation reactions were desalted and subjected to a one-pot desulfurization reaction using VA044, TCEP, and GSH to provide the desired singly acetylated analogs, **MaxK31Ac** and **MaxK57Ac**, in 33% and 37% isolated yields, respectively (see the SI, Section 5). Using the same synthetic protocol, we also prepared the fluorescently tagged version of the three acetylated Max analogs with 5-carboxytetramethylrhodamine (5-TAMRA), to afford the **T-MaxK31AcK57Ac**, **T-MaxK31Ac**, and **T-MaxK57Ac** analogs in the same manner (see the SI, Section 5). The analytical RP-HPLC and *m*/*z* spectra of the final products are depicted in Figure 3.

### The Acetylation of Lys 31 and Lys 57 Inhibits the DNA Binding Activity of Max

**Figure 4:**
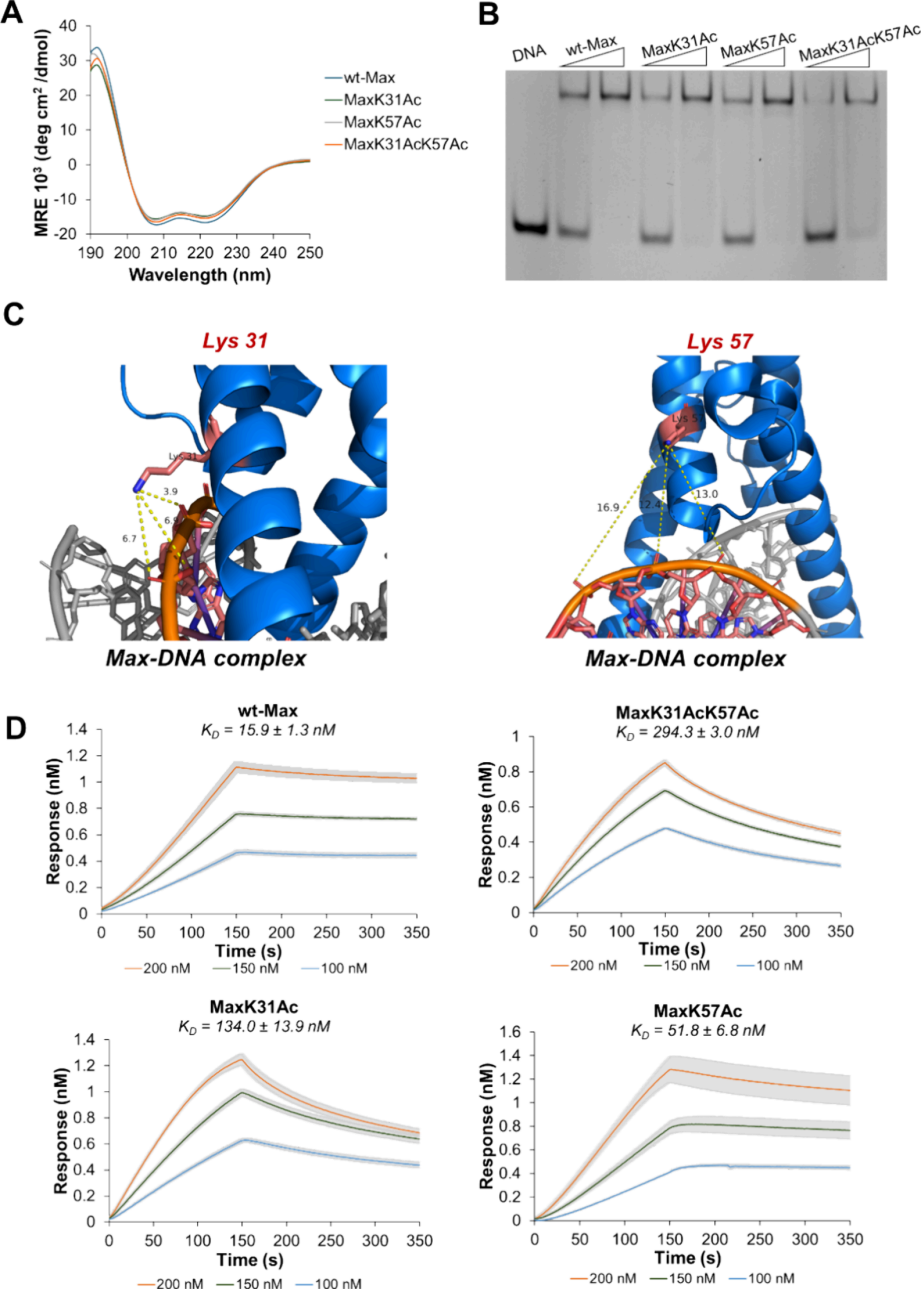
Max acetylation inhibits DNA binding of Max. (A) CD analysis of synthetic Max analogs; 10 μM protein was prepared in 10 mM MES, 150 mM KCl, 1 mM MgCl_2_, and 10% glycerol buffer (pH 6.0). The Max analogs exhibited a α-helical pattern as displayed by the deep double minima at 208 and 222 nm. Experiments were performed in triplicate. (B) EMSA experiment of synthetic Max analogs. Conditions: 1 μM DNA probe and 2 or 4 μM Max analogs incubated in 10 mM MES, 150 mM KCl, 1 mM MgCl_2_, and 10% glycerol buffer (pH 6.0) at room temperature. Experiments were performed in duplicate. (C) Illustration depicting the interaction of Lys residues with the phosphate diester in the DNA backbone (PDB: 1HLO).^8^ (D) Sensorgrams from biolayer interferometry (BLI) analysis of the binding of the synthetic Max analogs to the E-box DNA probe. Experiments were performed in triplicate. *K*_D_ values were calculated for each replicate, and the means and standard deviations derived from these three values are presented. Trend lines and error bars in these sensorgrams represent the aggregated data, showing the averages and standard deviations of each concentration and time point from the individual sensorgrams shown in Figure S19.

The acetylated Max analogs form homodimers and bind to the target E-box DNA with altered activity. We initially characterized the folding of the acetylated analogs **MaxK31Ac**, **MaxK57Ac**, and **MaxK31AcK57Ac** and the wild-type Max analog **wt-Max** using circular dichroism (CD) spectroscopy. In these experiments, all Max variants exhibited very similar α-helical structures, with deep double minima at 208 and 222 nm, as depicted in the CD spectrum (Figure 4A). Further, size-exclusion chromatography revealed that all synthetic Max variants efficiently dimerized to form the target 35 kDa Max homodimers with similar efficiency (SI, Section 6). Importantly, these results align with the solved X-ray structure of the Max complex, which shows that both acetylation sites, Lys 31 and Lys 57, are not part of the dimerizing interface of Max.^9,46^ Altogether, these results suggest that the acetylation marks do not interfere with the α-helical folding of Max or its homodimerization. To probe the impact of Lys-acetylation on the DNA binding activity of Max, we incubated each of the synthetic Max analogs separately with a double-stranded DNA probe containing the canonical E-box sequence and analyzed the DNA binding activity using an electrophoretic mobility shift assay (see EMSA, SI, Section 8). As a control, we used the native analogue **wt-Max**, which was efficiently associated with the E-box DNA probe, as indicated by an upward shift in a dose–response manner (Figure 4B). We observed a reduction in the DNA binding activity of all acetylated Max analogs compared with **wt-Max**, with the major inhibition mode obtained in the doubly acetylated analog **MaxK31AcK57Ac** (SI, Section 8). Comparing the monoacetylated analogs, we found that **MaxK31Ac** led to a slightly stronger reduction in the DNA binding activity compared with the other monoacetylated analog **MaxK57Ac** (SI, Section 8). These findings indicate that Max acetylation at Lys 31/57 plays a critical role in the DNA binding activity of Max potentially by interfering with essential interactions with the DNA. Interestingly, Lys 31 and Lys 57 residues are in the H1 and H2 α-helices of the HLH domain, and they are not part of the dimerizing interface of the HLH domain.^9,46^ Both residues have no direct contact with the consensus E-box nucleotides but are located proximal to the phosphate DNA backbone (Figure 4C). We envision that such a reduction in DNA binding activity could potentially result from the disruption of essential salt bridges between the Lys 31 and Lys 57 residues and the phosphate diester in the DNA backbone. Importantly, Lys 31 and Lys 57 are located approximately 3.9 and 12.4 Å from the DNA phosphate backbone (Figure 4C),^9,46^ which can potentially disrupt strategic interactions with the negatively charged phosphate backbone as a result of Lys residue acetylation and neutralization.

We then analyzed the reduction of the DNA binding activity by determining the dissociation constant of **wt-Max** and the doubly acetylated Max analog **MaxK31AcK57Ac** in relation to the E-box DNA probe via the biolayer interferometry (BLI) assay (see the SI, Section 9). We determined a *K*_D_ value of 15.9 ± 1.3 nM for **wt-Max**, which is in good agreement with previous reports for the recombinant Max (Figure 4D).^47^ Remarkably, we found about an 18-fold reduction in the dissociation constant value for the doubly acetylated analog **MaxK31AcK57Ac**, with a *K*_D_ value of 294.3 ± 3.0 nM. Importantly, we determined *K*_D_ values of 134.0 ± 13.9 nM and 51.8 ± 6.8 nM for the monoacetylated analogs **MaxK31Ac** and **MaxK57Ac**, respectively. Interestingly, these findings suggest that the acetylation of Lys 31 significantly inhibits DNA binding activity compared to Lys 57, with both acetylation sites contributing to the inhibition of the DNA binding activity of Max. Altogether, these findings reinforce that Lys-acetylation has a critical impact on the DNA binding activity of Max, leading to a reduction in the DNA binding activity potentially by neutralizing strategical Lys residues and destabilizing the TF–DNA complex formation.^48^

### The Acetylation Marks Alter the Binding Specificities of Max toward Certain Sequences Flanking Its Consensus Binding Sites

**Figure 5:**
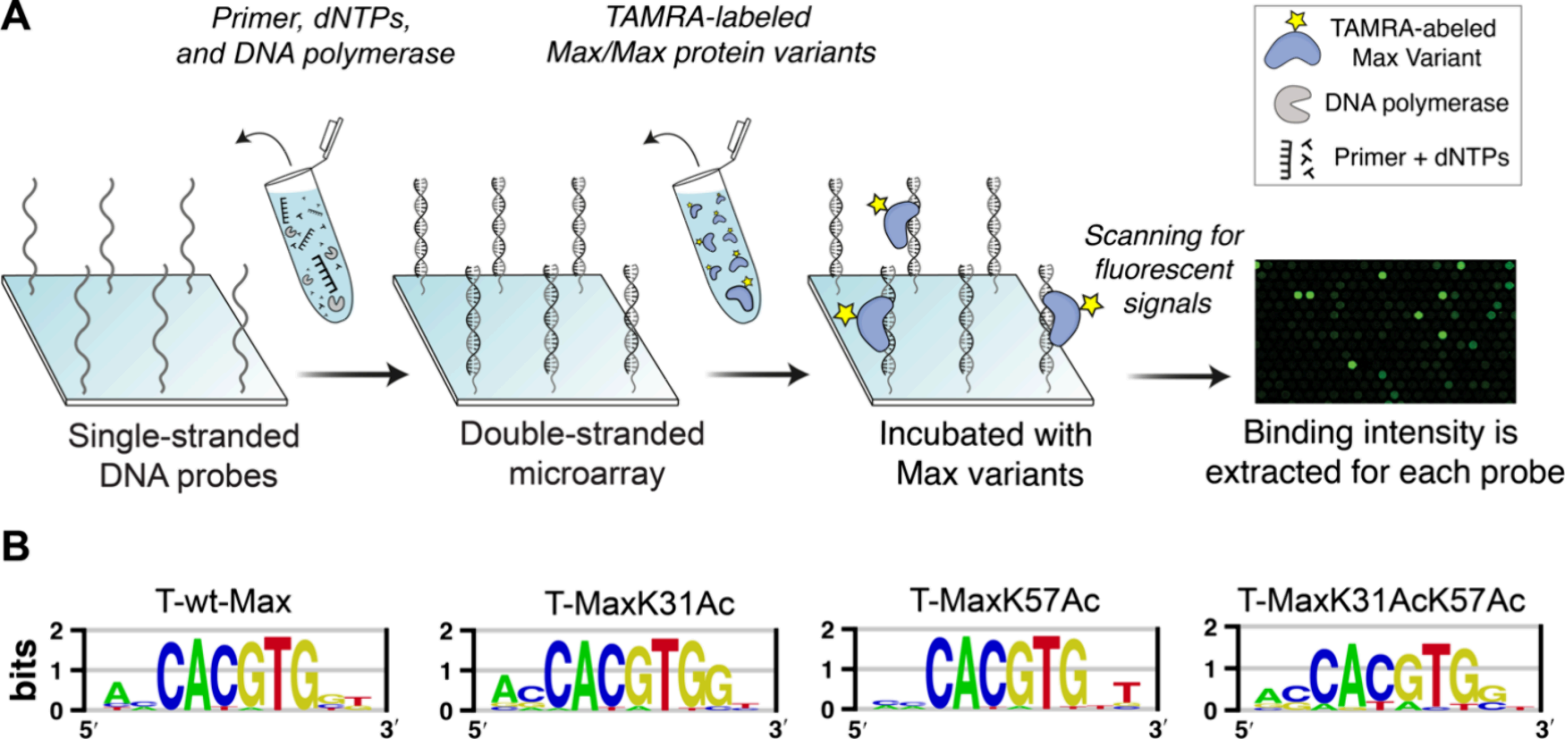
High-throughput universal protein binding microarray (PBM) experiments of synthetic Max variants show similar position weight matrix (PWM) logos for all four Max variants. (A) The protein binding microarray is a chip-based technology that simultaneously detects the binding of proteins to thousands of DNA sequences. The array encompasses all possible 10-mers and all contiguous and gapped 8-mers represented at least 32 times. The binding of TAMRA-labeled Max variants is quantified by capturing the fluorescent signals using a microarray scanner, which excites the samples at a 532 nm wavelength. (B) The PWM logos generated from the data of our PBM experiments with synthetic Max proteins show their preferred affinity to the E-box sequence (CACGTG) and confirm the functionality of all four Max variants.

The complex gene regulatory network within cells is governed by the binding of TFs to distinct genomic locations in a sequence-specific manner.^49^ Alterations in the sequence specificity of TFs can profoundly affect cellular activity, potentially contributing to disease development.^50^ Therefore, we aimed to elucidate how acetylation might shape Max’s recognition preferences across a broad spectrum of DNA sequences. For this purpose, we employed the protein binding microarray (PBM), a well-established high-throughput technique, which allows for simultaneous analysis of protein interactions with tens of thousands of DNA sequences on a single glass slide (see the SI, Section 11).^51,52^ The procedure starts by converting the commercially available single-stranded DNA microarrays to double-stranded DNA microarrays using Thermo Sequenase DNA polymerase and regular deoxynucleoside triphosphates (dNTPs). In the standard PBM protocol, the following steps usually involve introducing nonlabeled proteins and a fluorescent antibody to label them on the array, accompanied by multiple washing steps. We improved this process by directly introducing fluorescently labeled synthetic Max proteins,^53^ thereby eliminating the need for most of these steps. These fluorescent Max analogs enable the direct observation of protein–DNA interactions. The fluorescence emitted from these interactions was captured at a 532 nm wavelength with a resolution of 2.5 μm, and the intensity data for each probe on the array were gathered using GenePix software before it was normalized and analyzed (see the SI, Section 11). First, we conducted PBM experiments on a universal microarray that contains all possible DNA 10-mers in de-Bruijn sequences and generated a position weight matrix (PWM) logo based on the top enriched *k*-mer sequences.^52,53^ The PWM logos for the Max variants indicate a preference for binding to the CACGTG E-box sequence, confirming the functionality of all four synthetic Max variants. The general resemblance in the PWM logos underscores the common binding preferences across all variants and suggests that acetylation marks do not significantly impact the selectivity of the E-box core motif (Figure 5). A further examination of signals from all 8-mer sequences yields similar insights (see the SI, Figure S22). However, the subtle variations in the flanking region of the E-box prompted us to conduct a more in-depth analysis of the flanking preferences using an additional sequence library.

**Figure 6:**
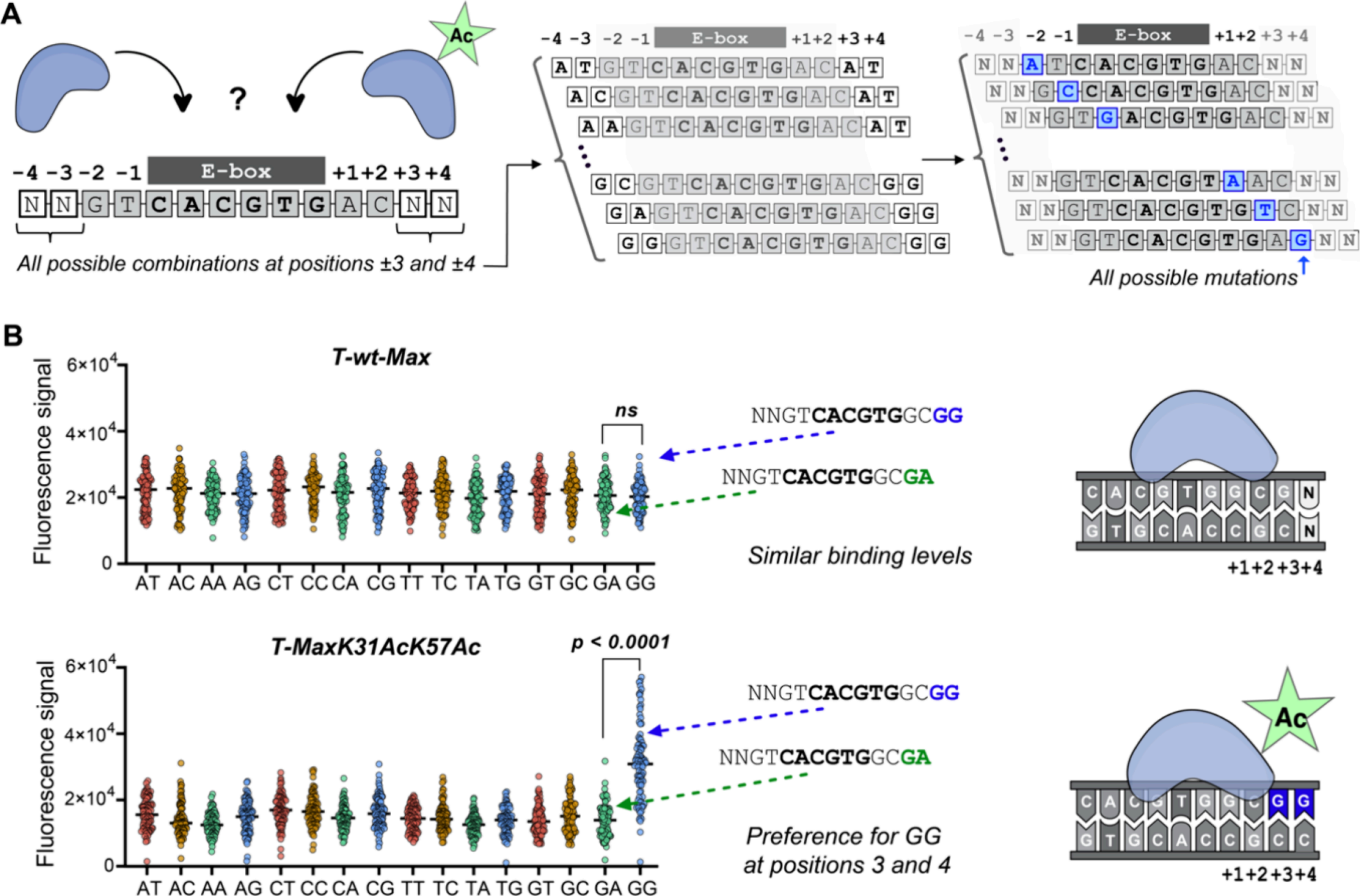
Analysis of binding specificities using custom sequence libraries reveals differential recognition by Max variants in the flanking regions of the E-box sequence (CACGTG). (A) Schematic illustration of the library design: To investigate the DNA binding preferences of synthetic Max variants at positions +3 and +4 relative to the core E-box motif (CACGTG), the library contains all possible base combinations at positions +3, +4, −3, and −4 (illustrated in the left and central panel). Additionally, it includes all possible single base pair (bp) variations within the E-box sequence and immediately adjacent to it at positions ±1 and ±2 (illustrated in the right panel). (B) The strip chart scatter plots illustrate the binding intensities of **T-wt-Max** (top panel) and **T-MaxK31AcK57Ac** (bottom panel) for a library of sequences. Each dot on the plots represents the median binding signal corresponding to a specific sequence. Although all sequences incorporate a consistent core, they diverge at the dinucleotide pairs at positions +3 and +4, which are labeled on the *x*-axis. **T-wt-Max** does not show a significant preference for any dinucleotide combination at these positions. In contrast, **T-MaxK31AcK57Ac** displays a statistically significant preference for the dinucleotide combination GG at positions +3 and +4 (two-tailed unpaired *t* test, *p* < 0.0001), implying that acetylation at lysine residues 31 and 57 leads to this change.

We therefore designed a custom microarray featuring a DNA sequence library that enabled us to explore selectivity at positions 3 and 4, situated farther from the E-box (see the SI, Figure S23). This library comprises all possible singly mutated 10-mer core sequences, in total, 25 core sequences, encompassing every conceivable combination of nucleotides at positions −4, −3, +3, and +4 (Figure 6A). Upon analysis of protein binding signals for the 16 possible nucleotide combinations at positions +3 and +4, **T-wt-Max**, **T-MaxK31Ac**, and **T-MaxK57Ac** revealed no distinct preference for a specific combination (see the SI, Figure S24). Surprisingly, in contrast to other variants, **T-MaxK31AcK57Ac** displays a robust preference for a subset of DNA sequences with ‘GG’ at positions +3 and +4 (Figure 6B, and SI, Figure S24). To further elucidate this selectivity, we isolated an outlier group. For this group with the 10-mer core sequence GTCACGTGGC, the binding signal intensity of **T-wt-Max** does not differ significantly for any specific dinucleotide combination at positions +3 and +4, as shown in Figure 6B (upper panel). In contrast, the binding signal intensity of **T-MaxK31AcK57Ac** toward sequences containing ‘GG’ at these positions is significantly higher (*t* test *p*-value <0.0001, Figure 6B, lower panel), and for some sequences, binding can be as much as three times higher compared to other combinations. We next conducted additional BLI measurements, replacing the ‘AT’ dinucleotides previously used at positions +3 and +4 (Figure 4) with ‘GG’ (see the SI, Figure S20). These experiments demonstrated that the **MaxK31AcK57Ac** variant indeed binds more efficiently to the ‘GG’ probe compared to the original ‘AT’ probes, with a 22% increase in binding (230 nM for ‘GG’ vs 294 nM for ‘AT’). This led us to question whether the **MaxK31AcK57Ac** variants could also exhibit these distinct preferences for genomic sequences containing the distal ‘GG’.

To address this, we included a library of several hundred genomic sequences obtained from ChIP-seq (chromatin immunoprecipitation followed by sequencing) data.^54^ These sites contain the core E-box sequence within different flanks, including various combinations of nucleotides at positions +3 and +4. We focused on the sequences with the highest binding signals (top 5%) for **T-wt-Max** and compared them with the top binders of **T-MaxK31AcK57Ac**. Remarkably, in agreement with our earlier findings, we identified a substantial enrichment of the ‘GG’ motif in the top binding sequences of **T-MaxK31AcK57Ac**, with a prevalence of around 28% of the top binding sequences. In contrast, the ‘GG’ pattern was notably absent from these positions in the **T-wt-Max** top sequences (see the SI, Figure S25). Similarly, whereas only 17% of the top sequences for **T-wt-Max** displayed a ‘G’ at position +4, this occurrence surged to approximately 61% in **T-MaxK31AcK57Ac**. These observations strongly support our earlier findings, suggesting that Lys acetylation may alter the DNA binding preferences of the Max protein within the genome, potentially influencing cellular functions. These findings highlight the intricate recognition patterns of post-translationally modified proteins, particularly in regions extending beyond the consensus binding sites.

## Conclusion

In this study, we employed total chemical protein synthesis and high-throughput technology to probe vital protein–DNA interactions and expand our understanding of how novel PTMs affect TF–DNA interactions to shed light on gene expression regulation events. We prepared site-specifically acetylated Max analogs from three peptide segments, which were ligated using sequential NCL reactions. This design enabled the production of homogeneous acetylated Max variants in good yield. Taking advantage of the synthetic analogs, we probed the impact of Lys acetylation on the DNA binding activity and sequence specificity of Max. Our results provide evidence that Lys acetylation at Lys 31 and Lys 57 significantly inhibits the DNA binding activity of Max TF. Both the Lys 31 and Lys 57 residues are proximal to the DNA phosphate backbone,^46^ thereby potentially interfering with the DNA binding activity of the Max complex upon acetylation by disrupting strategical interactions between Lys 31/57 with a phosphate diester in the DNA backbone. Furthermore, by employing high-throughput protein–DNA binding measurements for the various variants, we demonstrated that the acetylation mark can modify the binding preferences toward different sequences depending on the flanks of the core E-box binding motif. The influence of specific PTMs on key molecular interactions within the cell remains largely obscure. Abnormal acetylation levels have been linked to the onset of human diseases and the progression of cancer,^55^ including alterations attributed to the activity changes of p300/CBP,^56^ while p300 has been shown to acetylate Max in different levels,^15^ along with various other TFs,^57,58^ potentially explaining some of the observed changes in gene expression patterns in cancer and other human diseases. The precise influence of altering the acetylation marks of TFs’ binding activity in these diseases is yet to be explored. We anticipate this demonstration to prepare homogeneous synthetic TFs with the desired PTM pattern to pave the way for further studies to dissect crosstalk between Max-PTMs and other essential bHLH-TFs as well as design new analogs with refined activity.^59−65^ Importantly, given the different PTM levels and sites that are already identified in Max,^13−15,40^ a potential interplay between Max phosphorylation and acetylation can possibly represent an additional regulatory role in controlling the bHLH Myc/Max TF network, which requires further investigation. Finally, we envision that the synergy between total chemical synthesis and high-throughput technology will not only provide profound predictive insights into the impact of PTMs on the binding of additional oncogenic TFs to DNA but also lay the groundwork for comprehending other essential oligonucleotide-binding proteins of physiological significance. These and other research directions are currently being explored in our laboratory.

## Supporting Information Available

Synthesis and methods as well as characterization of all compounds (PDF)
