# Supplementary material for: Site-Specific Acetylation of the Transcription Factor Protein Max Modulates Its DNA Binding Activity
Source: ACS Cent Sci. 2024 Jun 12;10(6):1295–303. doi: 10.1021/acscentsci.4c00686 (PMC11212134; doi:10.1021/acscentsci.4c00686)
Supplement: Supplementary file 1 — oc4c00686_si_001.pdf [file oc4c00686_si_001.pdf]

# Supporting Information

## Site-specific Acetylation of the Transcription Factor Protein Max Modulates its DNA Binding Activity

Raj V. Nithun,[1+] Yumi Minyi Yao,[2+] Omer Harel,[1] Shaimaa Habiballah,[1] Ariel Afek,[2*] and Muhammad Jbara[1]*

[1]School of Chemistry, Raymond and Beverly Sackler Faculty of Exact Sciences, Tel Aviv University, Tel Aviv, 69978 Israel

[2]Department of Chemical and Structural Biology, Weizmann Institute of Science, Rehovot 7610001, Israel

[+]Contributed equally

*Correspondence to: ariel.afek@weizmann.ac.il, jbaram@tauex.tau.ac.il

# Table of Contents


**1. EXPERIMENTAL** .......... **3**

**1.1 MATERIALS** .......... 3
**1.2 ANALYTICAL HPLC-MS ANALYSIS** .......... 4
**1.3 PREPARATIVE RP-HPLC PURIFICATION** .......... 4
**1.4 pH MEASUREMENTS FOR LIGATION REACTIONS** .......... 4

**2. PROTEIN AND DNA SEQUENCES** .......... **5**

**3. PREPARATION OF 2-CHLOROTRITYL-HYDRAZINO-RESIN** .......... **5**

**4. CHEMICAL SYNTHESIS OF THE PEPTIDE SEGMENTS** .......... **6**

**4.1 SYNTHESIS OF SEGMENTS 1 CYS-MAX(93-151) & 1.1 CYS-MAX(93-151)-TAMRA** .......... 6
**4.2 SYNTHESIS OF SEGMENT 2.1 CYS-MAX(53-91)-NHNH$_2$** .......... 8
**4.3 SYNTHESIS OF SEGMENT 2.2 CYS-MAXK57AC(53-91)-NHNH$_2$** .......... 9
**4.4 SYNTHESIS OF SEGMENT 3.1 MAX(1-51)-NHNH$_2$** .......... 10
**4.5 SYNTHESIS OF SEGMENT 3.2 MAXK31AC(1-51)-NHNH$_2$** .......... 11

**5. CHEMICAL SYNTHESIS OF ACETYLATED MAX VARIANTS** .......... **13**

**5.1 NATIVE CHEMICAL LIGATION OF SEGMENTS 3.1 & 2.2** .......... 13
**5.2 NATIVE CHEMICAL LIGATION OF SEGMENTS 3.2 & 2.1** .......... 15
**5.3 NATIVE CHEMICAL LIGATION OF SEGMENTS 3.2 & 2.2** .......... 17
**5.4 CHEMICAL SYNTHESIS OF MAXK57AC VIA NATIVE CHEMICAL LIGATION-DESULFURIZATION METHOD** .......... 19
**5.5 CHEMICAL SYNTHESIS OF MAXK31AC VIA NATIVE CHEMICAL LIGATION-DESULFURIZATION METHOD** .......... 21
**5.6 CHEMICAL SYNTHESIS OF MAXK31ACK57AC VIA NATIVE CHEMICAL LIGATION-DESULFURIZATION METHOD** .......... 23
**5.7 CHEMICAL SYNTHESIS OF T-MAXK57AC VIA NATIVE CHEMICAL LIGATION-DESULFURIZATION METHOD** 25
**5.8 CHEMICAL SYNTHESIS OF T-MAXK31AC VIA NATIVE CHEMICAL LIGATION-DESULFURIZATION METHOD** 27
**5.9 CHEMICAL SYNTHESIS OF T-MAXK31ACK57AC VIA NATIVE CHEMICAL LIGATION-DESULFURIZATION METHOD** .......... 29
**5.10 CHEMICAL SYNTHESIS OF WT-MAX AND T-WT-MAX** .......... 31

**6. FOLDING AND ANALYSIS OF MAX VARIANTS** .......... **32**

**7. CIRCULAR DICHROISM (CD) ANALYSIS** .......... **33**

**8. DNA-BINDING ANALYSIS AND ELECTROPHORETIC MOBILITY-SHIFT ASSAY (EMSA)** .......... **33**

**9. OCTET BIOLAYER INTERFEROMETRY BINDING ASSAY (BLI)** .......... **36**

**10. ABSORPTION AND EMISSION SPECTRA OF TAMRA LABELED MAX ANALOGS** .......... **38**

**11. PROTEIN BINDING MICROARRAY ANALYSIS** .......... **39**

**12. REFERENCES** .......... **43**

# 1. Experimental

## 1.1 Materials

Fmoc-L-Phe-OH, Fmoc-L-Asn(Trt)-OH, Fmoc-L-Gln(Trt)-OH, Fmoc-L-Arg(Pbf)-OH, Fmoc-L-Tyr(tBu)-OH, Fmoc-L-Glu(OtBu)-OH, Fmoc-L-Ala-OH, Fmoc-L-Leu-OH, Fmoc-L-His(Trt)-OH, Fmoc-L-Asp(OtBu)-OH, Fmoc-L-Pro-OH, Fmoc-L-Cys(Trt)-OH, Fmoc-L-Lys(Boc)-OH, Fmoc-L-Lys(Alloc)-OH, Fmoc-L-Ile-OH, Fmoc-L-Ser(tBu)-OH, Fmoc-Gly-OH, Fmoc-L-Nle-OH, Boc-L-Nle-OH, Fmoc-L-Lys(Ac)-OH, Boc-L-Cys(Trt)-OH, Fmoc-L-Asp(OtBu)-(Dmb)Gly-OH, N,N´-Diisopropylcarbodiimide (DIC) and Ethidium bromide were purchased from Sigma-Aldrich. Fmoc-L-Ser(ψMe,Mepro)-OH was purchased from Iris-Biotech. Fmoc-L-His(Boc)-OH was purchased from CEM. 1-[Bis(dimethylamino)methylene]-1H-1,2,3-triazolo[4,5-b]pyridinium 3-oxid hexafluorophosphate (HATU), (2-(1H-benzotriazol-1-yl)-1,1,3,3-tetramethyluronium hexafluorophosphate (HBTU), HOBt HYDRATE were purchased from Luxembourg Bio Technologies Ltd. 2-Chlorotrityl chloride resin and Rink Amide ProTide (LL) resin were obtained from CEM. Oligonucleotides were purchased from Integrated DNA Technologies (IDT, Coralville, IA). Diethyl ether ($Et_2O$, 99.8% stabilized, ACS grade), was obtained from MACRON. Dichloromethane ($CH_2Cl_2$, ≥99.5% stabilized with 50 ppm Amylene) was obtained from CHEM-LAB. Peptide Synthesis-grade N,N-dimethylformamide (DMF) is from Fisher Scientific. Acetonitrile (LC/MS Grade) was purchased from J.T. Baker. Trifluoroacetic acid (TFA, ≥99% ReagentPlus®), diisopropylethylamine (DIEA, ≥99% ReagentPlus®), piperidine (≥99% ReagentPlus®), triisopropylsilane (TIS, 98%), formic acid (98-100% for LC/MS), and dimethyl sulfoxide (DMSO, ≥99.5% ReagentPlus®) were purchased from Bio-Lab ltd. 10% TBE Gel, (1.0mm x 10 well), TBE Running buffer (5X), and 6X DNA Loading Dye were purchased from Thermo Fisher Scientific. Water for all reactions carried out on proteins and for reverse-phase purification was obtained via filtration of deionized water through a MilliporeSigmaTM Milli-QTM Ultrapure Water System. All chemicals obtained from the supplier were used as received without further purification.

## 1.2 Analytical HPLC-MS analysis

Analytical HPLC were acquired using Thermo Scientific Vanquish HPLC, Mobile phases used are solvent A (0.05% formic acid in water) and solvent B (0.05% formic acid in acetonitrile) and mass spectrometry using Thermo Scientific LCQ Fleet Ion Trap Mass spectrometer.

Method A: bioZen™ 2.6 µm-C4 Widepore LC column (150 x 2.1 mm); LC conditions: 5% B from 0–1.0 min, then a linear gradient from 5% to 50% B from 1.0–11.0 min (i.e. 4.5% per min), 0.3 mL/min flow rate.

## 1.3 Preparative RP-HPLC purification

Preparative RP-HPLC was performed using Thermo Scientific DIONEX UltiMate 3000 Variable Wavelength Detector, equipped with a column of choice. Mobile phases used for LC analysis were solvent A (0.05% TFA in water), and solvent B (0.05% TFA in acetonitrile). The following LC methods were used:

Method A: Jupiter® 10 µm C18 300 Å LC column (250 x 21.2 mm), LC conditions: 5% B from 0–5 min, then a linear gradient from 5% to 40% B from 5-40 min (i.e. 1% per min), 25 mL/min flow rate at RT.

Method B: XBridge® Protein BEH C4 OBD™ Prep column, 300 Å, 5 µm, (250 x 10 mm), LC conditions: 5% B from 0–5 min, then a linear gradient from 5% to 20% B from 5-10 min, followed by another linear gradient from 20% to 50% B from 10-55 min (i.e. 0.67% per min), 4 mL/min flow rate at 30 °C.

Method C: Jupiter® 5 µm C4 300 Å LC column (250 x 10 mm), LC conditions: 5% B from 0–5 min, then a linear gradient from 5% to 60% B from 5-60 min (i.e. 1% per min), 4 mL/min flow rate at 30 °C.

Method D: XBridge® Protein BEH C4 OBD™ Prep column, 300 Å, 5 µm, (250 x 10 mm), LC conditions: 5% B from 0–5 min, then a linear gradient from 5% to 40% B from 5-40 min (i.e. 1% per min), 4 mL/min flow rate at 30 °C.

Method E: XBridge® Protein BEH C4 OBD™ Prep column, 300 Å, 5 µm, (250 x 10 mm), LC conditions: 5% B from 0–5 min, then a linear gradient from 5% to 20% B from 5-10 min, followed by another linear gradient from 20% to 60% B from 10-50 min (i.e. 1% per min), 4 mL/min flow rate at 30 °C.

## 1.4 pH measurements for ligation reactions

All pH values in aqueous 6 M Gn.HCl were determined using a VWR pH meter with a SENTEK electrode.

## 2. Protein and DNA sequences

**Max(1-151):**

MSDNDDIEVESDADKRAHHNALERKRRDHI**K**$^{31}$DSFHSLRDSVPSLQGEKASRAQILD**K**$^{57}$ATEYIQYMRRKNHTHQQDIDDLKRQNALLEQQVRALEKARSSAQLQTNYPSSDNSLYTNAKGSTISAFDGGSDSSSESEPEEPQSRKKLRMEAS-*K*

*The Met residue at positions 1, 65 and 148 were replaced with the isologous norleucine (Nle) residue to avoid Met oxidation.*

**E-box DNA probe:**

E-box-1 5'-CCGGCTGA*CACGTG*GTATTAAT-3'

E-box-2 5'-CCGGCTGA*CACGTG*GTGGTAAT-3'

## 3. Preparation of 2-chlorotrityl-hydrazino-resin

The preparation was carried out by the following scheme.

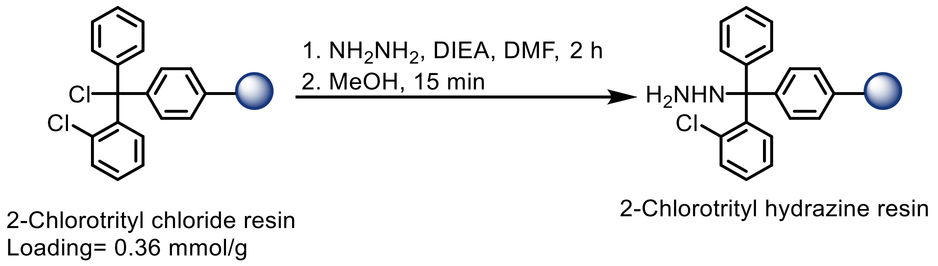


286 mg 2-Chlorotrityl chloride resin (loading = 0.36 mmol/g, 0.1 mmol) was swelled in DMF for 15 min and cooled to 0 °C. A mixture of DIEA (190 µl, 1.1 mmol, 1.3 M) and hydrazine hydrate-50% (96 µl, 3.1 mmol, 3.6 M) in DMF (571 µl) was added slowly. Then, the suspension was stirred at room temperature. After 2 h, 115 µl MeOH was added to the reaction mixture to ensure the unreacted sites on the resin are capped and stirred for an additional 15 min. Finally, the resin was washed with DMF, $H_2O$, DMF, MeOH, and $Et_2O$ and dried under a vacuum.[1]

# 4. Chemical synthesis of the peptide segments

## 4.1 Synthesis of segments 1 Cys-Max(93-151) & 1.1 Cys-Max(93-151)-TAMRA

The synthesis was carried out according to the following scheme:

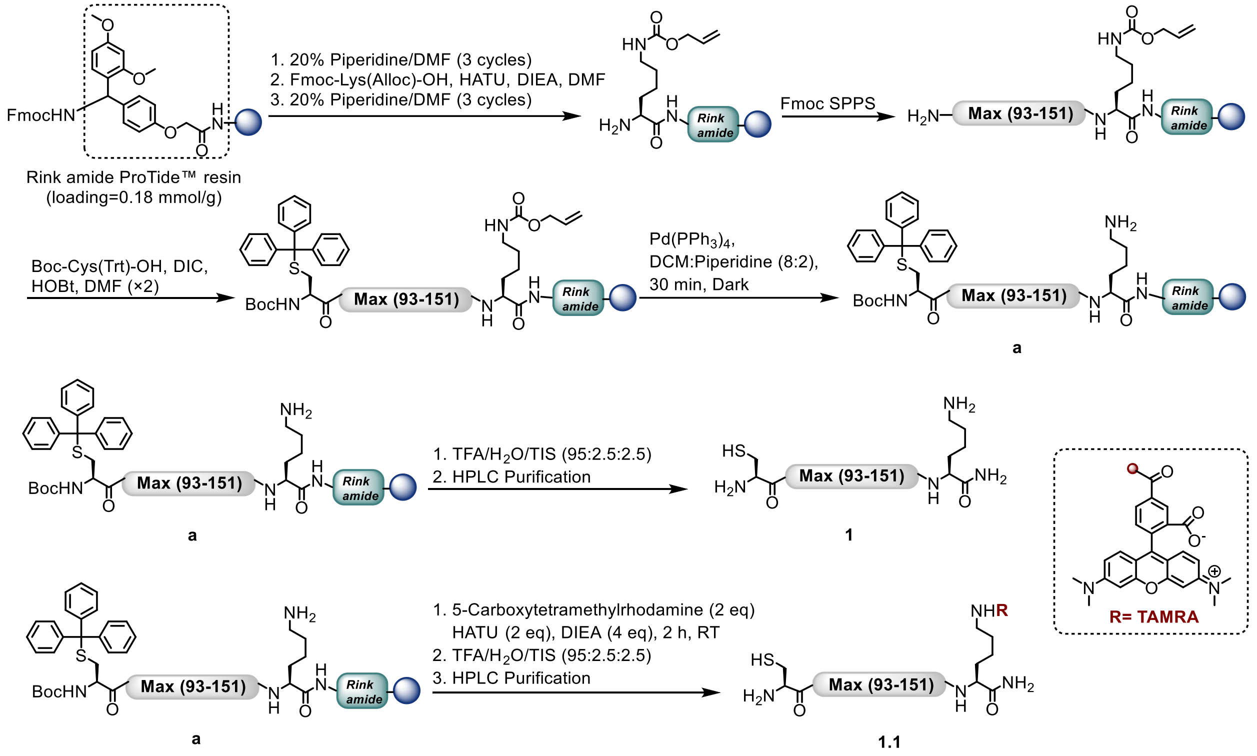


The synthesis of peptide segments **1** and **1.1** was carried out using stepwise Fmoc chemistry SPPS on Rink amide ProTide™ resin (556 mg, loading 0.18 mmol/g, 0.1 mmol scale). The resin was pre-swelled in DMF for 30 min and then treated with 20% piperidine, 0.05% formic acid in DMF to remove the Fmoc protecting group, subsequently the functionalized resin was coupled with Fmoc-Lys(Alloc)-OH (4 equiv., 0.4 mmol, 0.19 M), using HATU (4 equiv., 0.4 mmol, 0.19 M) and 0.15 mL DIEA (0.86 mmol, 0.4 M) in 2 mL DMF for 2 h. The resin was then transferred to the CSBio automated peptide synthesizer and residues 151-128 added in stepwise fashion with Fmoc-amino acid (10 equiv., 1 mmol, 66.7 mM), using HBTU/HOBt (10 equiv., 1 mmol, 66.7 mM) and 0.37 mL DIEA (2 mmol, 133.3 mM) per amino acid. The coupling was carried out at room temperature (RT). To the resin was manually coupled Fmoc-L-Asp(OtBu)-(Dmb)Gly-OH dipeptide (2.5 equiv., 0.25 mmol, 0.12 M) using HATU (2.5 equiv., 0.25 mmol, 0.12 M) and 0.08 mL DIEA (0.46 mmol, 0.22 M) in 2 mL DMF for 2 h. The resin was again transferred to the CSBio automated peptide synthesizer for continued stepwise addition of Fmoc-amino acids in a similar manner through amino acid residue 99. Residue 98 was coupled as Fmoc-L-Ser(ψMe,Mepro)-OH (5 equiv., 0.5 mmol, 0.23 M) using HBTU/HOBt (5 equiv., 0.5 mmol, 0.23 M) and 0.18 mL DIEA (1 mmol, 0.46 M). The remaining

amino acids were coupled manually with Fmoc-amino acid (10 equiv., 1 mmol, 0.42 M), using HBTU/HOBt (10 equiv., 1 mmol, 0.42 M) and 0.37 mL DIEA (2 mmol, 0.84 M) in 2 mL DMF for 45 min. The last amino acid Boc-L-Cys(Trt)-OH (5 equiv., 0.5 mmol, 0.24 M) was coupled twice with HOBt/DIC (5 equiv., 0.5 mmol, 0.24 M/5 equiv., 0.5 mmol, 0.24 M). Finally, to remove the Alloc protecting group the resin was washed with DCM (5 mL x 3) and treated with $Pd(PPh_3)_4$ (1 equiv., 0.1 mmol) in DCM/piperidine (8:2, 4 ml) and shaken for 30 min at 25 °C under exclusion of light. The resin was split, and half was coupled with 5-Carboxytetramethylrhodamine (TAMRA, 2 equiv., 0.1 mmol, 50 mM) with HATU (2 equiv., 0.1 mmol, 50 mM) and DIEA (0.2 mmol, 0.1 M) for 2 h at RT. After that, both peptide resins were washed with DMF (5 mL x 3), MeOH (5 mL x 3), and DCM (5 mL x 3) and dried under vacuum. To remove side chain protecting groups and release the peptide chains, a mixture of TFA/$H_2O$/TIS (95:2.5:2.5, 7 mL for 0.025 mmol scale) was added to each peptide resin and shaken for 3.5 h at RT. The resin was removed by filtration and washed with TFA (2 × 1 mL). To precipitate the peptide, the combined filtrate was added dropwise to cold diethyl ether (25 mL for 0.025 mmol resin) followed by centrifugation at 4000 rpm for 7 min. Then, the diethyl ether was decanted, followed by the dissolution of the peptide in 50% acetonitrile/water, diluted to 25% acetonitrile/water, and lyophilized to get crude segment **1** Cys-Max(93-151) (284 mg, 43.2 µmol) as white powder and crude segment **1.1** Cys-Max(93-151)-TAMRA (258 mg, 37.0 µmol) as red powder. The crude dry peptide powder was purified by RP-HPLC (Method A for segment **1** and Method B for segment **1.1** described in Section 1.3) affording the product Cys-Max(93-151) (39 mg, 5.9 µmol, 12% yield based on 0.05 mmol resin) as a white powder and Cys-Max(93-151)-TAMRA (31 mg, 4.4 µmol, 9% yield based on 0.05 mmol resin) as a red powder.

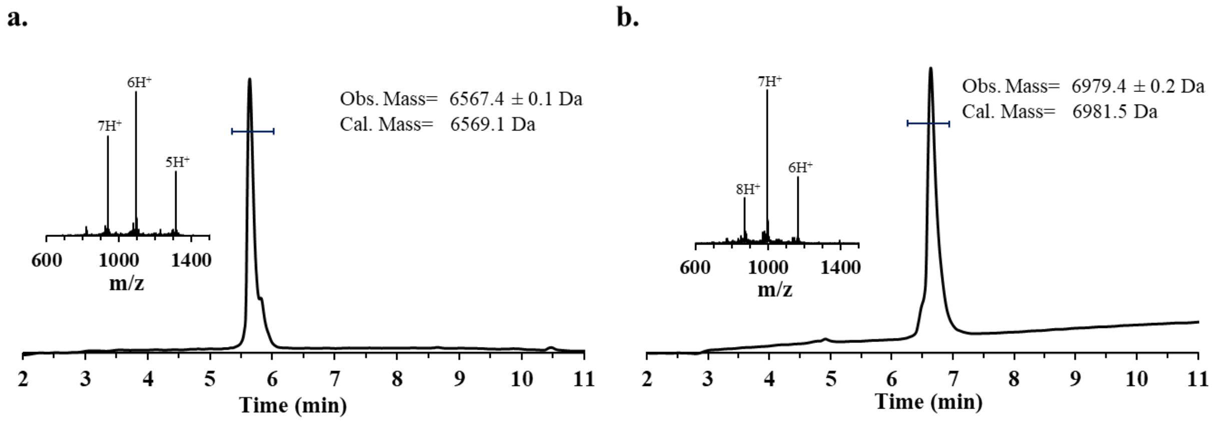


**Figure S1. (a)** Analytical HPLC-MS analysis of segment **1** Cys-Max(93-151) with the observed mass 6567.4 ± 0.1 Da, calculated mass 6569.1 Da (average isotopes) and **(b) 1.1** Cys-Max(93-151)-TAMRA with the observed mass 6979.4 ± 0.2 Da, calculated mass 6981.5 Da (average isotopes). The UV absorbance was monitored at 214 nm and the mass-to-charge (m/z) data acquired over the marked region in the chromatogram. HPLC-MS analysis was carried out with Method A depicted in section 1.2 (5-50% acetonitrile gradient; 4.5% per min).

## 4.2 Synthesis of segment 2.1 Cys-Max(53-91)-NHNH$_2$

The synthesis was carried out according to the following scheme:

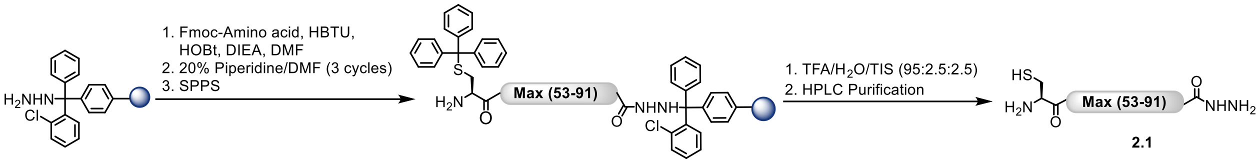


The synthesis of the segment **2.1** Cys-Max(53-91)-NHNH$_2$ was carried out using stepwise Fmoc chemistry SPPS on hydrazide resin (139 mg, loading 0.36 mmol/g, 0.05 mmol scale). The resin was pre-swelled in DMF for 30 min and then transferred to the CSBio automated peptide synthesizer and residues 91-52 added in a stepwise fashion with Fmoc-amino acid (10 equiv., 0.5 mmol, 33.3 mM), using HBTU/HOBt (10 equiv., 0.5 mmol, 33.3 mM) and 0.18 mL DIEA (20 equiv., 1 mmol, 66.7 mM). The coupling was carried out at 60 °C for 15 minutes coupling time. When the synthesis was completed, the peptide resin was washed with DMF (5 mL x 3), MeOH (5 mL x 3), and DCM (5 ml x 3) and dried under vacuum. To remove side chain protecting groups and release the peptide chains, a mixture of TFA/$H_2O$/TIS (95:2.5:2.5, 7 mL for 0.025 mmol scale) was added to the resin which was shaken for 3 h at RT. The resin was removed by filtration and washed with TFA (2 × 1 mL). To precipitate the peptide, the combined filtrate was added dropwise to cold diethyl ether (25 mL for 0.025 mmol resin) followed by centrifugation at 4000 rpm for 7 min. Then, the diethyl ether was decanted, followed by the dissolution of the peptide in 25% acetonitrile/water and lyophilized to get crude segment **2.1** Cys-Max(53-91)-NHNH$_2$ (205 mg, 41.5 µmol) as white powder. The crude dry peptide powder was purified by RP-HPLC (Method C described in Section 1.3) affording the product Cys-Max(53-91)-NHNH$_2$ (47 mg, 9.5 µmol, 19% yield based on 0.05 mmol resin) as a white powder.

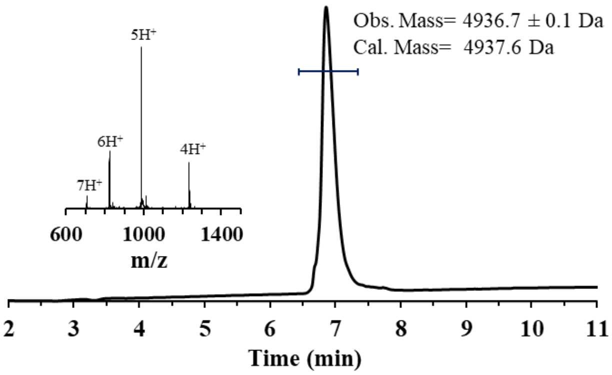


**Figure S2.** Analytical HPLC-MS analysis of segment **2.1** Cys-Max(53-91)-NHNH$_2$ with the observed mass 4936.7 ± 0.1 Da, calculated mass 4937.6 Da (average isotopes). The UV absorbance was monitored at 214 nm and the m/z data acquired over the marked region in the chromatogram. HPLC-MS analysis was carried out with Method A depicted in section 1.2 (5-50% acetonitrile gradient; 4.5% per min).

## 4.3 Synthesis of segment 2.2 Cys-MaxK57Ac(53-91)-$NHNH_2$

The synthesis was carried out according to the following scheme:

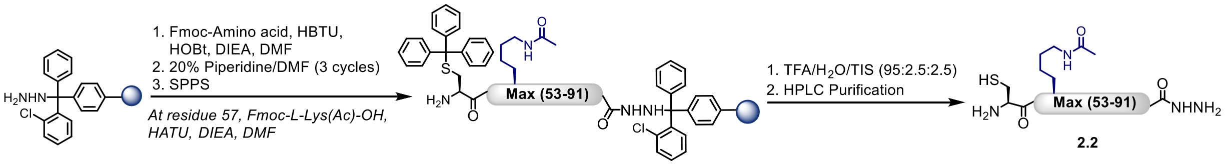


The synthesis of the segment **2.2** Cys-MaxK57Ac(53-91)-$NHNH_2$ was carried out using stepwise Fmoc chemistry SPPS on hydrazide resin (139 mg, loading 0.36 mmol/g, 0.05 mmol scale). The resin was pre-swelled in DMF for 30 min and then transferred to the CSBio automated peptide synthesizer and residues 91-58 added in a stepwise fashion with Fmoc-amino acid (10 equiv., 0.5 mmol, 33.3 mM), using HBTU/HOBt (10 equiv., 0.5 mmol, 33.3 mM) and 0.18 mL DIEA (20 equiv., 1 mmol, 66.7 mM). The coupling was carried out at 60 °C for 15 minutes coupling time. To the resin was manually coupled Fmoc-Lys(Ac)-OH (5 equiv., 0.25 mmol, 0.12 M) using HATU (5 equiv., 0.25 mmol, 0.12 M) and 90 µl DIEA (10 equiv., 0.5 mmol, 0.24 M) in 2 mL DMF for 1 h. The resin was again transferred to the CSBio automated peptide synthesizer and continued step wise addition of the remaining amino acids in a similar manner. When the synthesis was completed, the peptide resin was washed with DMF (5 mL x 3), MeOH (5 mL x 3), and DCM (5 ml x 3) and dried under vacuum. To remove side chain protecting groups and release the peptide chains, a mixture of TFA/$H_2O$/TIS (95:2.5:2.5, 7 mL for 0.025 mmol scale) was added to the resin which was shaken for 3 h at RT. The resin was removed by filtration and washed with TFA (2 × 1 mL). To precipitate the peptide, the combined filtrate was added dropwise to cold diethyl ether (25 mL for 0.025 mmol resin) followed by centrifugation at 4000 rpm for 7 min. Then, the diethyl ether was decanted, followed by the dissolution of the peptide in 25% acetonitrile/water and lyophilized to get crude Cys-MaxK57Ac(53-91)-$NHNH_2$ (231 mg, 46.4 µmol) as white powder. The crude dry peptide powder was purified by RP-HPLC (Method C described in Section 1.3) affording the product Cys-MaxK57Ac(53-91)-$NHNH_2$ (45 mg, 9.0 µmol, 18% yield based on 0.05 mmol resin) as a white powder.

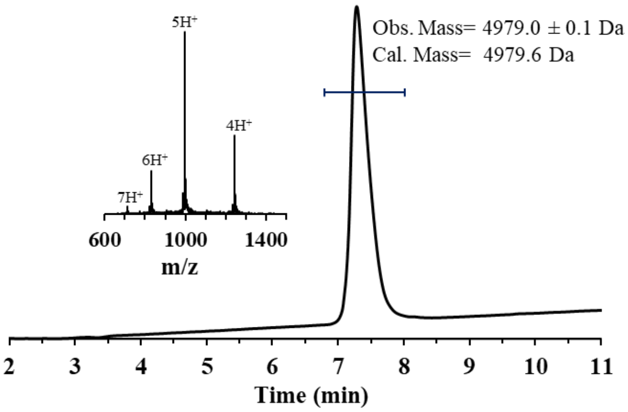


**Figure S3.** Analytical HPLC-MS analysis of segment **2.2** Cys-MaxK57Ac(53-91)-$NHNH_2$ with the observed mass 4979.0 ± 0.1 Da, calculated mass 4979.6 Da (average isotopes). The UV absorbance was monitored at 214 nm and the m/z data acquired over the marked region in the chromatogram. HPLC-MS analysis was carried out with Method A depicted in section 1.2 (5-50% acetonitrile gradient; 4.5% per min).

### 4.4 Synthesis of segment 3.1 Max(1-51)-$NHNH_2$

The synthesis was carried out according to the following scheme:

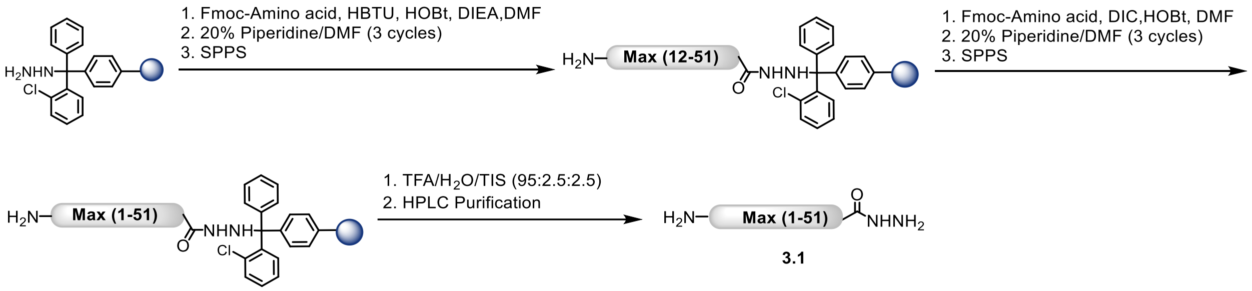


The synthesis of segment **3.1** Max(1-51)-$NHNH_2$ was carried out using stepwise Fmoc chemistry SPPS on hydrazide resin (139 mg, loading 0.36 mmol/g, 0.05 mmol scale). The resin was pre-swelled in DMF for 30 min and then transferred to the CSBio automated peptide synthesizer and residues 51-12 added in a stepwise fashion with Fmoc-amino acid (10 equiv., 0.5 mmol, 33.3 mM), using HBTU/HOBt (10 equiv., 0.5 mmol, 33.3 mM) and 0.18 mL DIEA (20 equiv., 1 mmol, 66.7 mM). Residues 11-1 was then added with Fmoc-amino acid (10 equiv., 0.5 mmol, 33.3 mM) using DIC (10 equiv., 0.5 mmol, 33.3 mM) and HOBt (10 equiv., 0.5 mmol, 33.3 mM) in the same way. All coupling were carried out at 30 °C for 45 minutes coupling time. When the synthesis was completed, the peptide resin was washed with DMF (5 mL x 3), MeOH (5 mL x 3), and DCM (5 ml x 3) and dried under vacuum. To remove side chain protecting groups and release the peptide chains, a mixture of TFA/$H_2O$/TIS (95:2.5:2.5, 7 mL for 0.025 mmol scale) was added to the resin which was shaken for 3.5 h at RT. The resin was removed by filtration and washed with TFA (2 × 1 mL). To precipitate the peptide, the combined filtrate was added dropwise to cold diethyl

ether (25 mL for 0.025 mmol resin) followed by centrifugation at 4000 rpm for 7 min. Then, the diethyl ether was decanted, followed by the dissolution of the peptide in 25% acetonitrile/water and lyophilized to get crude Max(1-51)-$NHNH_2$ (260 mg, 44.1 µmol) as white powder. The crude dry peptide powder was purified by RP-HPLC (Method D described in Section 1.3) affording the product Max(1-51)-$NHNH_2$ (49 mg, 8.3 µmol, 17% yield based on 0.05 mmol resin) as a white powder.

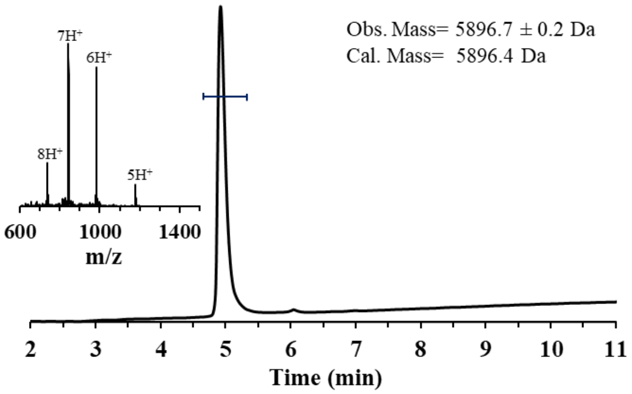


**Figure S4.** Analytical HPLC-MS analysis of segment **3.1** Max(1-51)-$NHNH_2$ with the observed mass 5896.7 ± 0.2 Da, calculated mass 5896.4 Da (average isotopes). The UV absorbance was monitored at 214 nm and the m/z data acquired over the marked region in the chromatogram. HPLC-MS analysis was carried out with Method A depicted in section 1.2 (5-50% acetonitrile gradient; 4.5% per min).

### 4.5 Synthesis of segment 3.2 MaxK31Ac(1-51)-$NHNH_2$

The synthesis was carried out according to the following scheme:

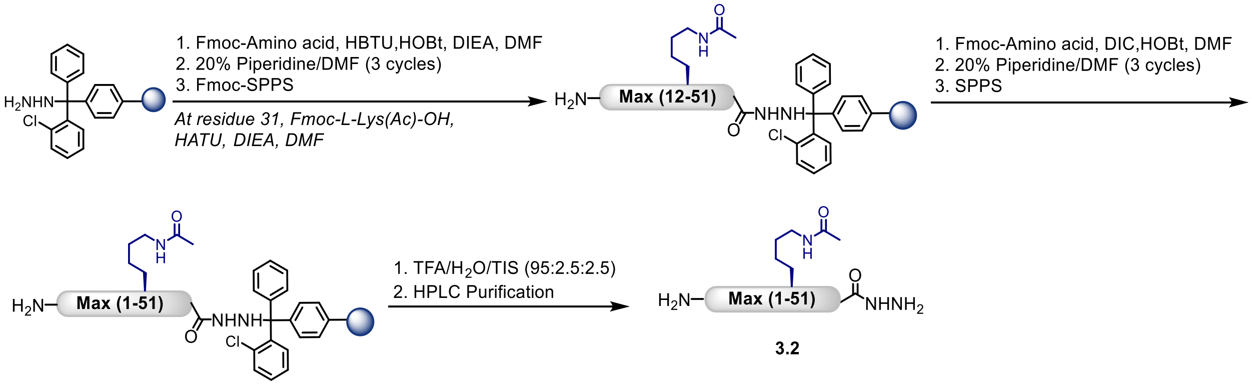


The synthesis of segment **3.2** MaxK31Ac(1-51)-$NHNH_2$ was carried out using stepwise Fmoc chemistry SPPS on hydrazide resin (278 mg, loading 0.36 mmol/g, 0.1 mmol scale). The resin was pre-swelled in DMF for 30 min and then transferred to the CSBio automated peptide synthesizer and residues 51-32 added in a stepwise fashion with Fmoc-amino acid (10 equiv., 1 mmol, 66.7 mM), using HBTU/HOBt (10 equiv., 1 mmol, 66.7 mM) and 0.37 mL DIEA (2 mmol, 0.13 M). The coupling was carried out at 30 °C for 45

minutes coupling time. To the resin was manually coupled Fmoc-Lys(Ac)-OH (5 equiv., 0.5 mmol, 0.23 M) using HATU (5 equiv., 0.5 mmol, 0.23 M) and 0.18 mL DIEA (10 equiv., 1 mmol, 0.46 M) in 2 mL DMF for 1 h. The resin was again transferred to the CSBio automated peptide synthesizer and continued step wise addition of the remaining amino acids in a similar manner till residue 12. Residues 11-1 was added with Fmoc-amino acid (10 equiv., 1 mmol, 66.7 mM) using DIC (10 equiv., 1 mmol, 66.7 mM) and HOBt (10 equiv., 1 mmol, 66.7 mM) in the same way using CSBio automated peptide synthesizer. When the synthesis was completed, the peptide resin was washed with DMF (5 mL x 3), MeOH (5 mL x 3), and DCM (5 ml x 3) and dried under vacuum. To remove side chain protecting groups and release the peptide chains, a mixture of TFA/$H_2O$/TIS (95:2.5:2.5, 7 mL for 0.025 mmol scale) was added to the resin which was shaken for 3 h at RT. The resin was removed by filtration and washed with TFA (2 × 1 mL). To precipitate the peptide, the combined filtrate was added dropwise to cold diethyl ether (25 mL for 0.025 mmol resin) followed by centrifugation at 4000 rpm for 7 min. Then, the diethyl ether was decanted, followed by the dissolution of the peptide in 25% acetonitrile/water and lyophilized to get crude MaxK31Ac(1-51)-$NHNH_2$ (236 mg, 39.7 µmol) as white powder. The crude dry peptide powder was purified by RP-HPLC (Method D described in Section 1.3) affording the product MaxK31Ac(1-51)-$NHNH_2$ (44 mg, 7.4 µmol, 15% yield based on 0.05 mmol resin) as a white powder.

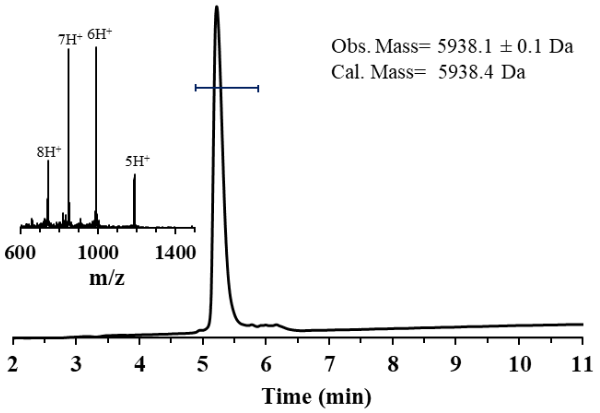


**Figure S5.** Analytical HPLC-MS analysis of segment **3.2** MaxK31Ac(1-51)-$NHNH_2$ with the observed mass 5938.1 ± 0.1 Da, calculated mass 5938.4 Da (average isotopes). The UV absorbance was monitored at 214 nm and the m/z data acquired over the marked region in the chromatogram. HPLC-MS analysis was carried out with Method A depicted in section 1.2 (5-50% acetonitrile gradient; 4.5% per min).

# 5. Chemical synthesis of acetylated Max variants

## 5.1 Native chemical ligation of segments 3.1 & 2.2

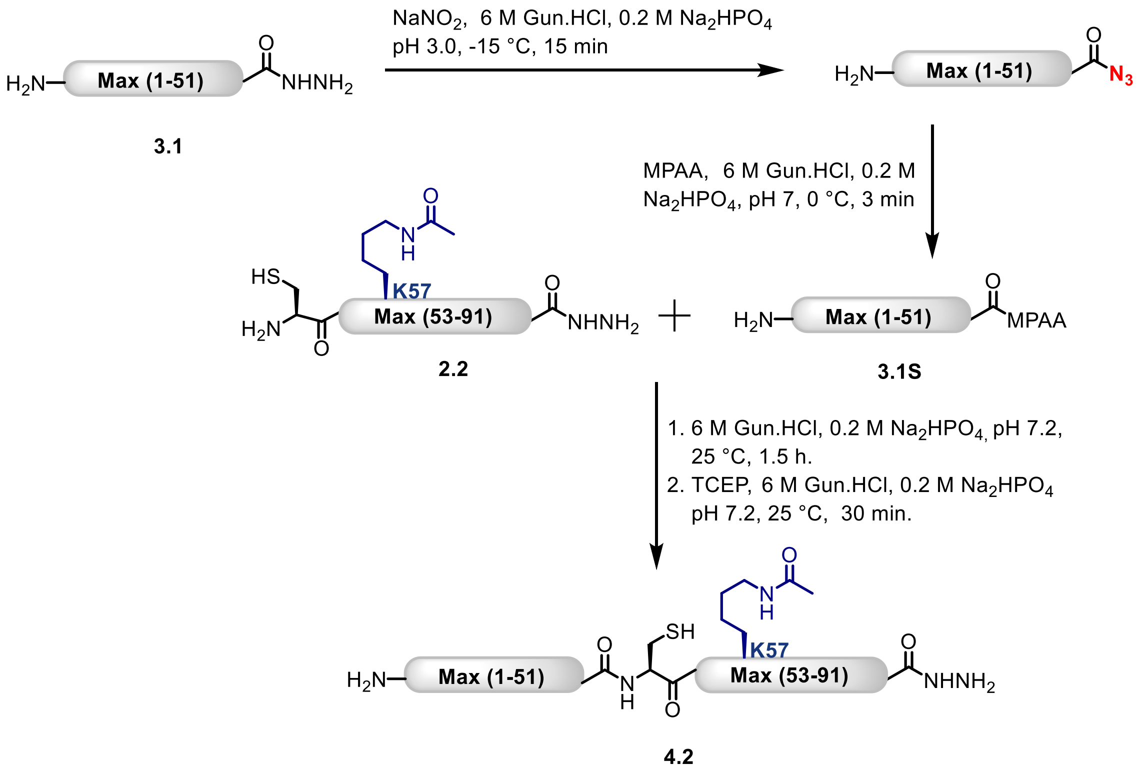


Segment **3.1** Max(1-51)-$NHNH_2$ (8 mg, 1.4 equiv., 1.36 µmol, 9 mM) was dissolved in 6 M Gun.HCl, 0.2 M $Na_2HPO_4$ buffer (150 µL) at pH 3.0 and cooled down to -15 °C by placing in an ice/salt bath. 10 µL of $NaNO_2$ (10 equiv., 13.6 µmol, 1.36 M; based on **3.1**) dissolved in water was added to the reaction mixture and allowed to react for 15 min at -15 °C with gentle mixing in repeated intervals. After 15 min 150 µL of MPAA (50 equiv., 68 µmol, 0.45 M; based on **3.1**) in 6 M Gun.HCl, 0.2 M $Na_2HPO_4$ buffer at pH 7 was added to the mixture and gently mixed for two-three minutes. Then, segment **2.2** Cys-MaxK57Ac(53-91)-$NHNH_2$ (4.8 mg, 1.0 equiv., 0.96 µmol, 3.1 mM) was dissolved in the reaction mixture and the pH was adjusted to 7.2 using 1 *N* NaOH at 0 °C. The mixture was then incubated for 1.5 h at 25 °C and then 150 µL of TCEP (40 equiv., 54.4 µmol, 0.36 M; based on **3.1**) in 6 M Gun.HCl, 0.2 M $Na_2HPO_4$ buffer at pH 7.2 was added and continued incubating for 30 min at 25 °C. The reaction was monitored using analytical HPLC-MS (Method A described in Section 1.2).[2,3] After 2 h ligation, the reaction mixture was diluted with $H_2O$ and purified using preparative RP-HPLC (Method E described in Section 1.3) to afford 7.6 mg (0.7 µmol) of MaxK57Ac(1-91)-$NHNH_2$ **4.2** as a white powder (73% yield, based on the limiting segment **2.2**).

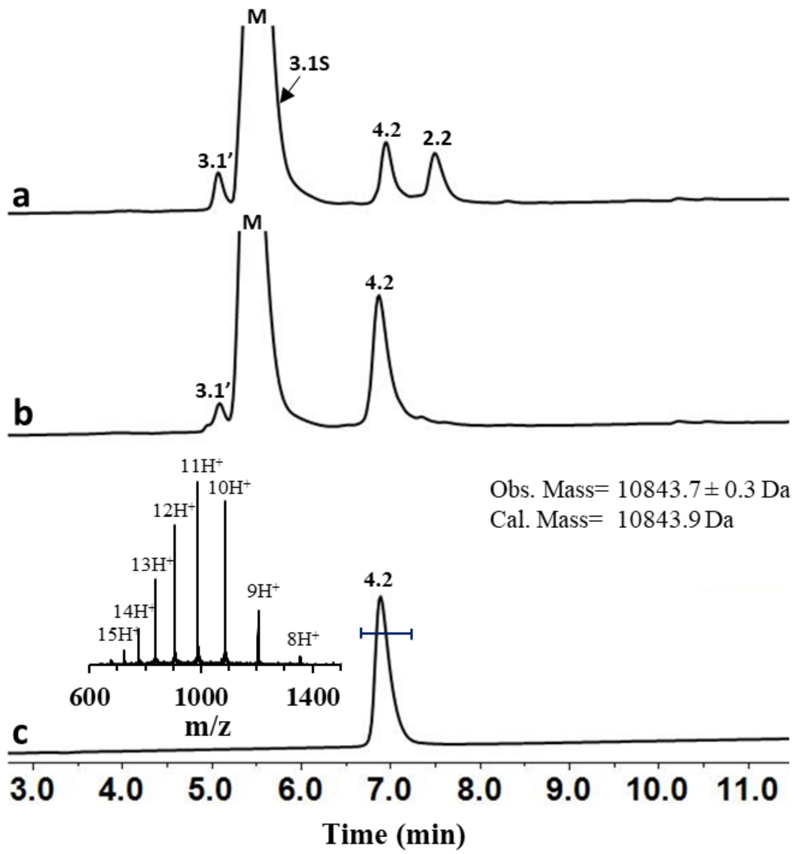


**Figure S6.** Analytical HPLC-MS of the progress of Max(1-51)-$NHNH_2$ (**3.1**) and Cys-MaxK57Ac(53-91)-$NHNH_2$ (**2.2**) ligation. **(a)** Ligation at t = 0 min; Max(1-51) thioester segment (**3.1S**), hydrolysis of Max(1-51) thioester (**3.1'**), ligated product MaxK57Ac(1-91)-$NHNH_2$ (**4.2**), and M=MPAA. **(b)** Crude ligation reaction at t = 120 min. **(c)** Ligated product MaxK57Ac(1-91)-$NHNH_2$ (**4.2**) after purification with the observed mass 10843.7 ± 0.3 Da, calculated mass 10843.9 Da (average isotopes). The UV absorbance was monitored at 214 nm and the m/z data acquired over the marked region in the chromatogram.

## 5.2 Native chemical ligation of segments 3.2 & 2.1

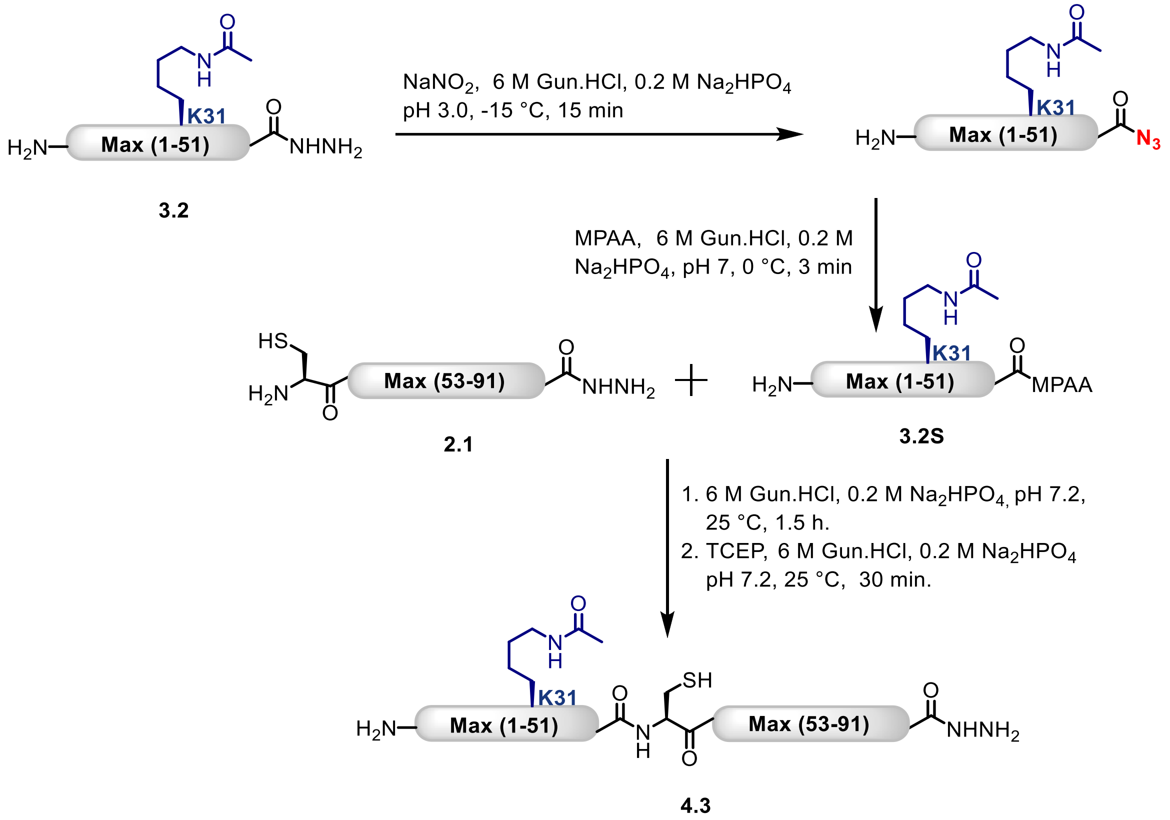


Segment **3.2** MaxK31Ac(1-51)-NHNH$_2$ (8 mg, 1.4 equiv., 1.35 µmol, 9 mM) was dissolved in 6 M Gun.HCl, 0.2 M $Na_2HPO_4$ buffer (150 µL) at pH 3.0 and cooled down to -15 °C by placing in an ice/salt bath. 10 µL of $NaNO_2$ (10 equiv., 13.6 µmol, 1.35 M; based on **3.2**) dissolved in water was added to the reaction mixture and allowed to react for 15 min at -15 °C with gentle mixing in repeated intervals. After 15 min 150 µL of MPAA (50 equiv., 67.5 µmol, 0.45 M; based on **3.2**) in 6 M Gun.HCl, 0.2 M $Na_2HPO_4$ buffer at pH 7 was added to the mixture and gently mixed for two-three minutes. The segment **2.1** Cys-Max(53-91)-NHNH$_2$ (4.8 mg, 1 equiv., 0.97 µmol, 3.1 mM) was then dissolved in the reaction mixture and the pH was adjusted to 7.2 using 1 *N* NaOH at 0 °C. The mixture was then incubated for 1.5 h at 25 °C and then 150 µL of TCEP (40 equiv., 54 µmol, 0.36 M; based on **3.2**) in 6 M Gun.HCl, 0.2 M $Na_2HPO_4$ buffer at pH 7.2 was added and continued incubating for 30 min at 25 °C. The reaction was monitored using analytical HPLC-MS (Method A described in Section 1.2). After 2 h ligation, the reaction mixture was diluted with $H_2O$ and purified using preparative RP-HPLC (Method E described in Section 1.3) to afford 5.8 mg (0.53 µmol) of MaxK31Ac(1-91)-NHNH$_2$ **4.3** as a white powder (55% yield, based on the limiting segment **2.1**).

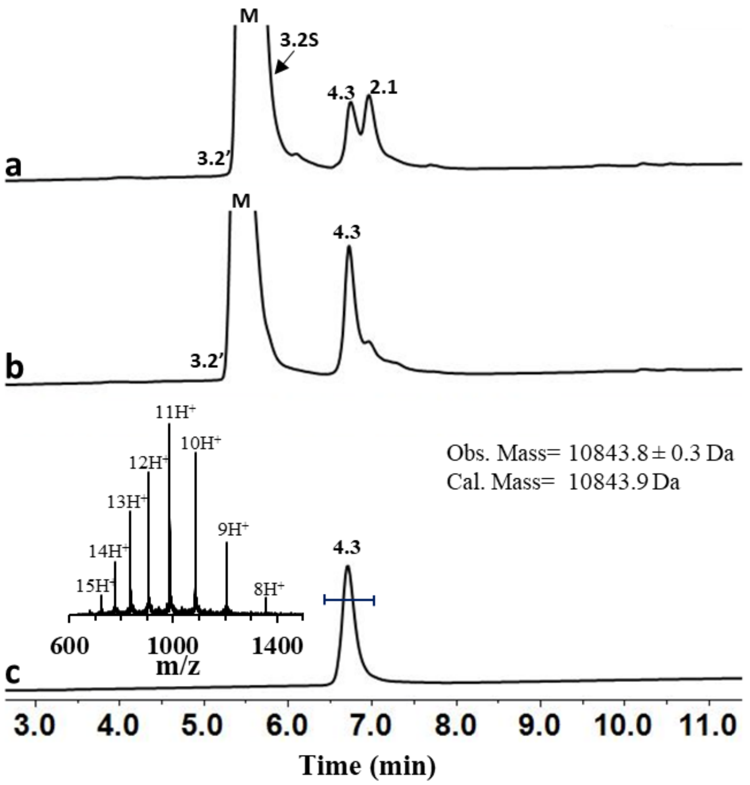


**Figure S7.** Analytical HPLC-MS of the progress of MaxK31Ac(1-51)-$NHNH_2$ (**3.2**) and Cys-Max(53-91)-$NHNH_2$ (**2.1**) ligation. **(a)** Ligation at t = 0 min; MaxK31Ac(1-51) thioester segment (**3.2S**), hydrolysis of MaxK31Ac(1-51) thioester (**3.2'**), ligated product MaxK31Ac(1-91)-$NHNH_2$ (**4.3**), and M=MPAA. **(b)** Crude ligation reaction at t = 120 min. **(c)** Ligated product MaxK31Ac(1-91)-$NHNH_2$ (**4.3**) after purification with the observed mass 10843.8 ± 0.3 Da, calculated mass 10843.9 Da (average isotopes). The UV absorbance was monitored at 214 nm and the m/z data acquired over the marked region in the chromatogram.

## 5.3 Native chemical ligation of segments 3.2 & 2.2

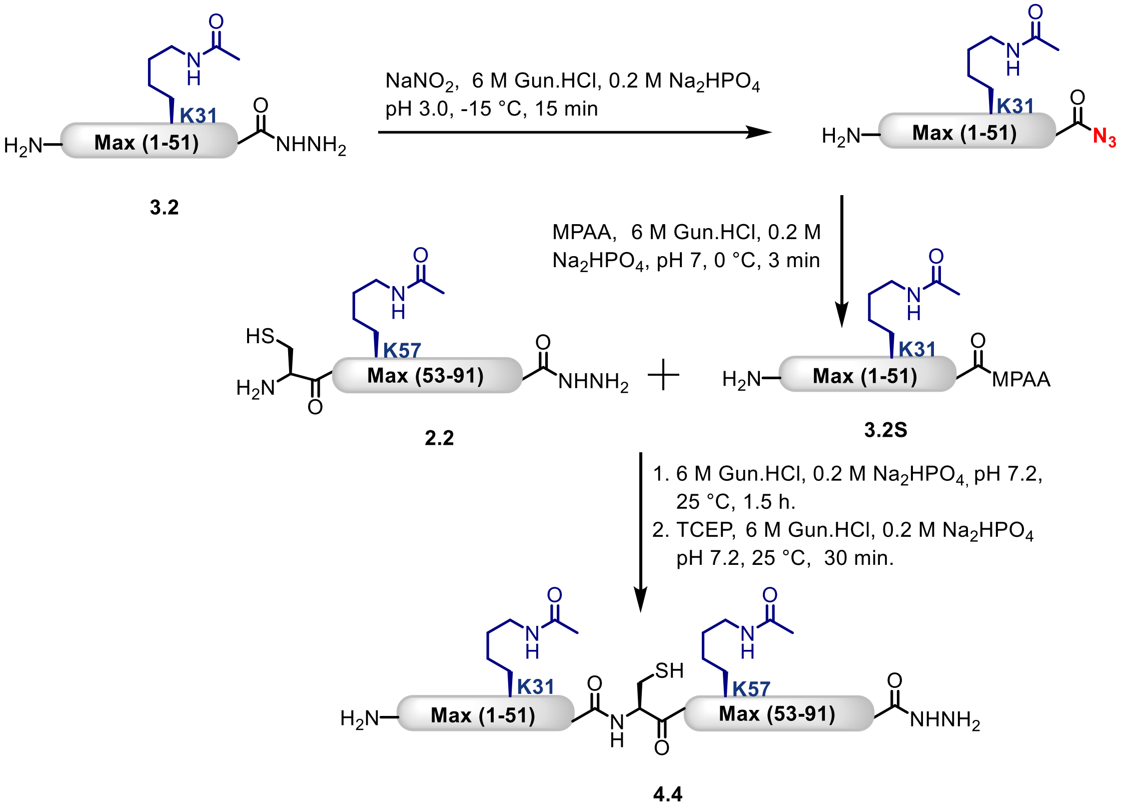


Segment **3.2** MaxK31Ac(1-51)-NHNH$_2$ (6.7 mg, 1.4 equiv., 1.13 µmol, 7.5 mM) was dissolved in 6 M Gun.HCl, 0.2 M $Na_2HPO_4$ buffer (150 µL) at pH 3.0 and cooled down to -15 °C by placing in an ice/salt bath. 10 µL of $NaNO_2$ (10 equiv., 11.3 µmol, 1.13 M; based on **3.2**) dissolved in water was added to the reaction mixture and allowed to react for 15 min at -15 °C with gentle mixing in repeated intervals. After 15 min 150 µL of MPAA (50 equiv., 56.5 µmol, 0.38 M; based on **3.2**) in 6 M Gun.HCl, 0.2 M $Na_2HPO_4$ buffer at pH 7 was added to the mixture and gently mixed for two-three minutes. The segment **2.2** Cys-MaxK57Ac(53-91)-NHNH$_2$ (4 mg, 1 equiv., 0.8 µmol, 2.6 mM) was then dissolved in the reaction mixture and the pH was adjusted to 7.2 using 1 *N* NaOH at 0 °C. The mixture was then incubated for 1.5 h at 25 °C and then 150 µL of TCEP (40 equiv., 45.2 µmol, 0.3 M; based on **3.2**) in 6 M Gun.HCl, 0.2 M $Na_2HPO_4$ buffer at pH 7.2 was added and continued incubating for 30 min at 25 °C. The reaction was monitored using analytical HPLC-MS (Method A described in Section 1.2). After 2 h ligation, the reaction mixture was diluted with $H_2O$ and purified using preparative RP-HPLC (Method E described in Section 1.3) to afford 5.9 mg (0.54 µmol) of MaxK31AcK57Ac(1-91)-NHNH$_2$ **4.4** as a white powder (68% yield, based on the limiting segment **2.2**).

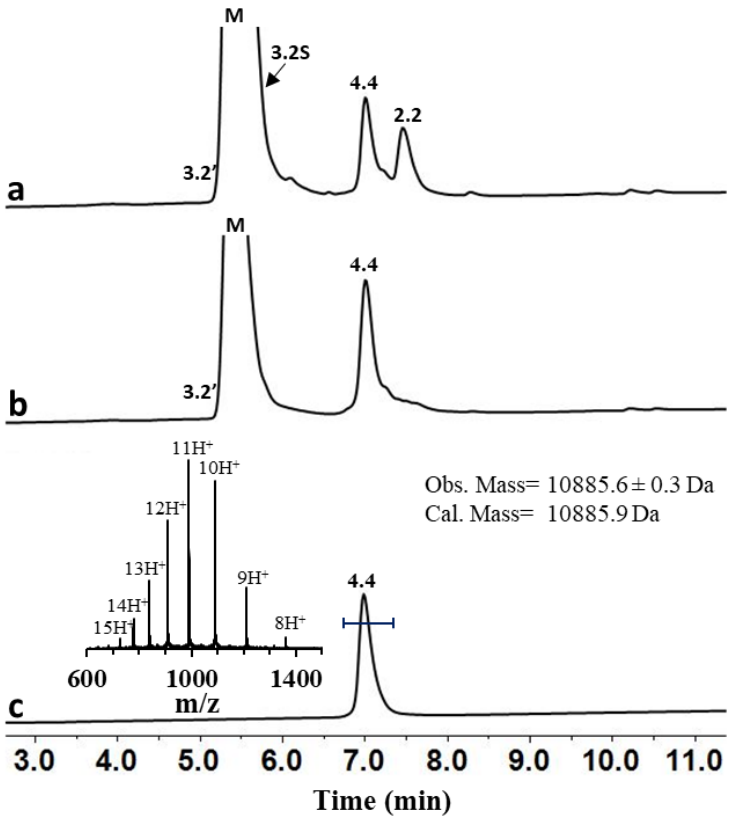


**Figure S8.** Analytical HPLC-MS of the progress of MaxK31Ac(1-51)-$NHNH_2$ (**3.2**) and Cys-MaxK57Ac(53-91)-$NHNH_2$ (**2.2**) ligation. **(a)** Ligation at t = 0 min; MaxK31Ac(1-51) thioester segment (**3.2S**), hydrolysis of MaxK31Ac(1-51) thioester (**3.2'**), ligated product MaxK31AcK57Ac(1-91)-$NHNH_2$ (**4.4**), and M=MPAA. **(b)** Crude ligation reaction at t = 120 min. **(c)** Ligated product MaxK31AcK57Ac(1-91)-$NHNH_2$ (**4.4**) after purification with the observed mass 10885.6 ± 0.3 Da, calculated mass 10885.9 Da (average isotopes). The UV absorbance was monitored at 214 nm and the m/z data acquired over the marked region in the chromatogram.

### 5.4 Chemical synthesis of MaxK57Ac via native chemical ligation-desulfurization method

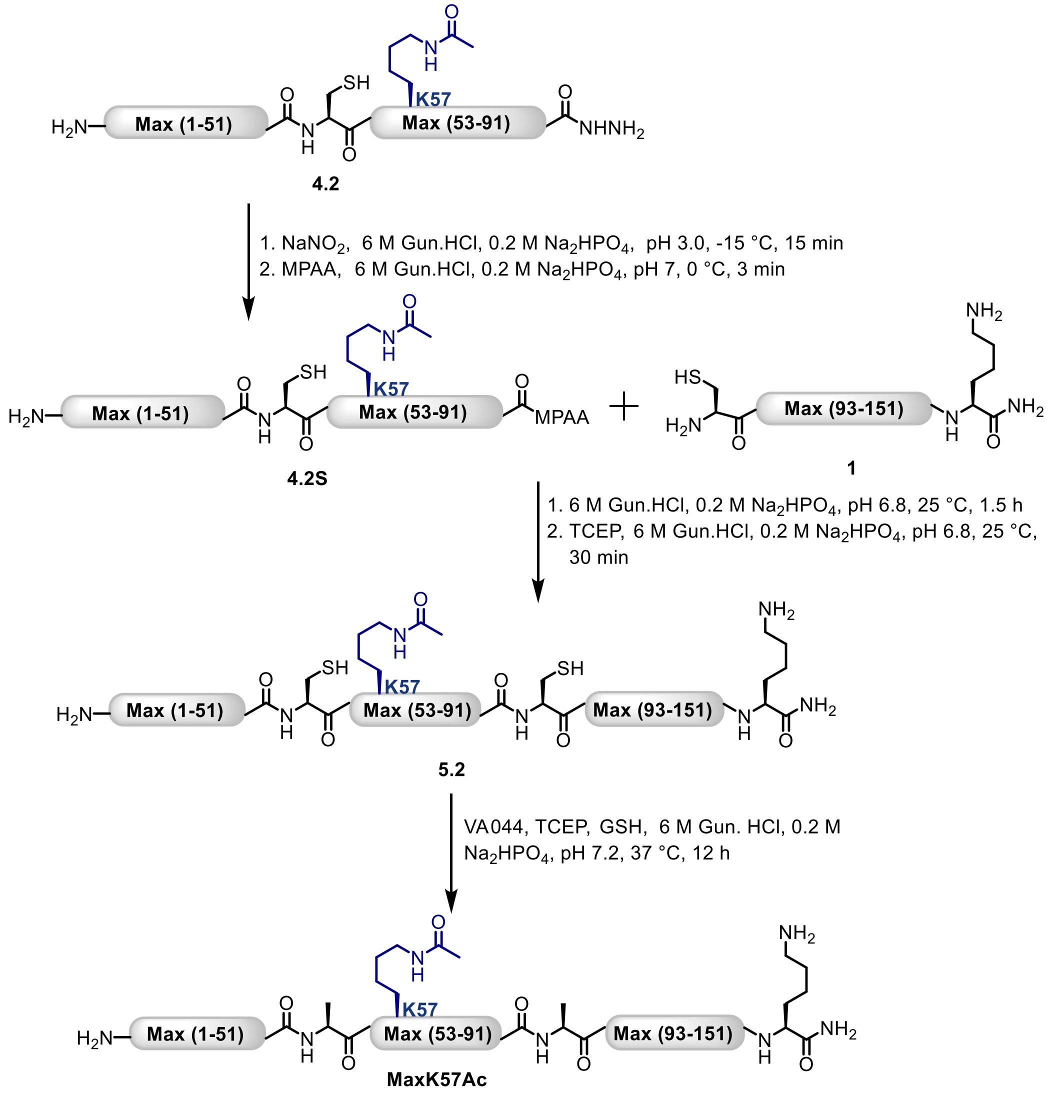


MaxK57Ac(1-91)-NHNH2 **4.2** (4.6 mg, 1.4 equiv., 0.42 µmol, 5.6 mM) was dissolved in a 6 M Gun.HCl, 0.2 M $Na_2HPO_4$ buffer (75 µL) at pH 3.0 and cooled down to -15 °C by placing in an ice/salt bath. 5 µL of $NaNO_2$ (10 equiv., 4.2 µmol, 0.84 M; based on **4.2**) dissolved in water was added to the reaction mixture and allowed to react for 15 min at -15 °C with gentle mixing in repeated intervals. After 15 min 75 µL of MPAA (50 equiv., 21 µmol, 0.28 M; based on **4.2**) in 6 M Gun.HCl, 0.2 M $Na_2HPO_4$ buffer at pH 7 was added to the mixture and gently mixed for two-three minutes. The segment **1** Cys-Max(93-151) (2 mg, 1 equiv., 0.3 µmol, 2 mM) was then dissolved in the reaction mixture and the pH was adjusted to 6.8 using 1 *N* NaOH at 0 °C. The mixture was then incubated for 1.5 h at 25 °C and then 75 µL of TCEP (40 equiv., 16.8 µmol, 0.22 M; based on **4.2**) in 6 M Gun.HCl, 0.2 M $Na_2HPO_4$ buffer at pH 6.8 was added and continued incubating for 30 min at 25 °C. The reaction was monitored using analytical HPLC-MS (Method A described in Section 1.2). The ligation was completed in 2 h. After completion of the reaction, the crude

reaction was desalted by pipetting the reaction mixture into a 10 kDa molecular weight cutoff spin filter (Amicon® Ultra- 2mL, 10K). The reaction mixture was diluted with a 6 M Gun.HCl, 0.2 M $Na_2HPO_4$ buffer (pH 7.2) to 2.0 mL and concentrated to 1.0 mL by Centrifuging the spin filter at 5000 rpm for 15 min. This process was repeated four more times. In the final process, the reaction mixture was concentrated into 400 µL. After that, the reaction mixture was collected by reverse centrifuge and then treated with VA044 (80 µmol, 200 mM), TCEP (0.1 mmol, 250 mM), and L-Glutathione (GSH, 24 µmol, 60 mM) for 12 h.[4,5] The progress of the reaction was monitored by analytical HPLC-MS using Method A (Section 1.2). After the completion of the reaction, purification was carried out using RP-HPLC (Method E described in Section 1.3) affording 1.9 mg (0.11 µmol) of the final product **MaxK57Ac** as a white powder (37% yield, based on the limiting segment **1**).

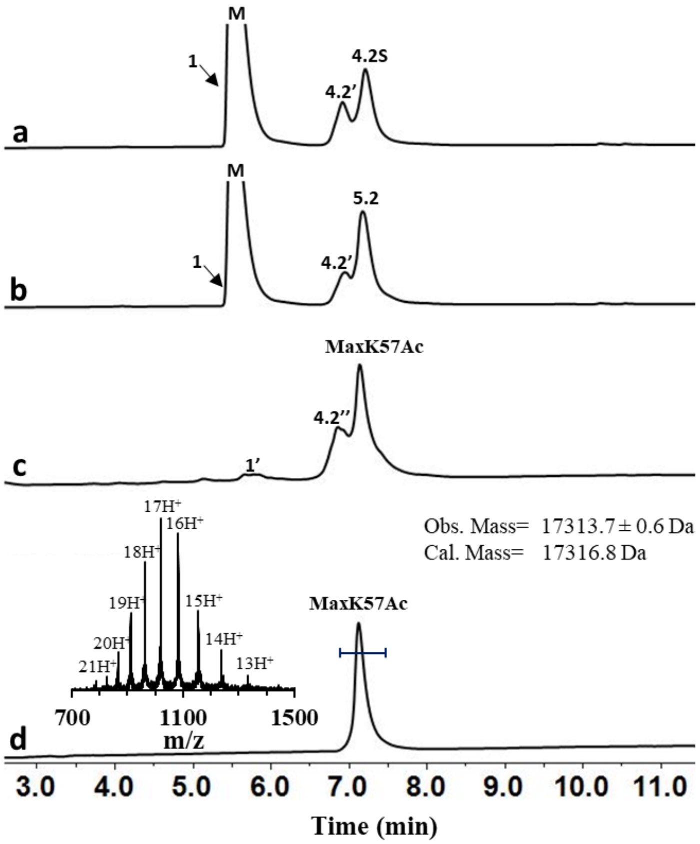


**Figure S9.** Analytical HPLC-MS of the progress of the native chemical ligation and one-pot desulfurization. **(a)** Ligation at t = 0 min; MaxK57Ac(1-91) thioester segment (**4.2S**), hydrolysis of MaxK57Ac(1-91) thioester (**4.2'**), Cys-Max(93-151) segment (**1**), and M=MPAA. **(b)** Crude ligation reaction at t = 120 min; ligated product **5.2** **(c)** Crude desulfurization reaction at t = 12 h; desulfurized segment **1** (**1'**), desulfurized-**4.2'** (**4.2''**) **(d)** RP-HPLC purified **MaxK57Ac** with the observed mass 17313.7 ± 0.6 Da, calculated mass 17316.8 Da (average isotopes). The UV absorbance was monitored at 214 nm and the m/z data acquired over the marked region in the chromatogram.

### 5.5 Chemical synthesis of MaxK31Ac via native chemical ligation-desulfurization method

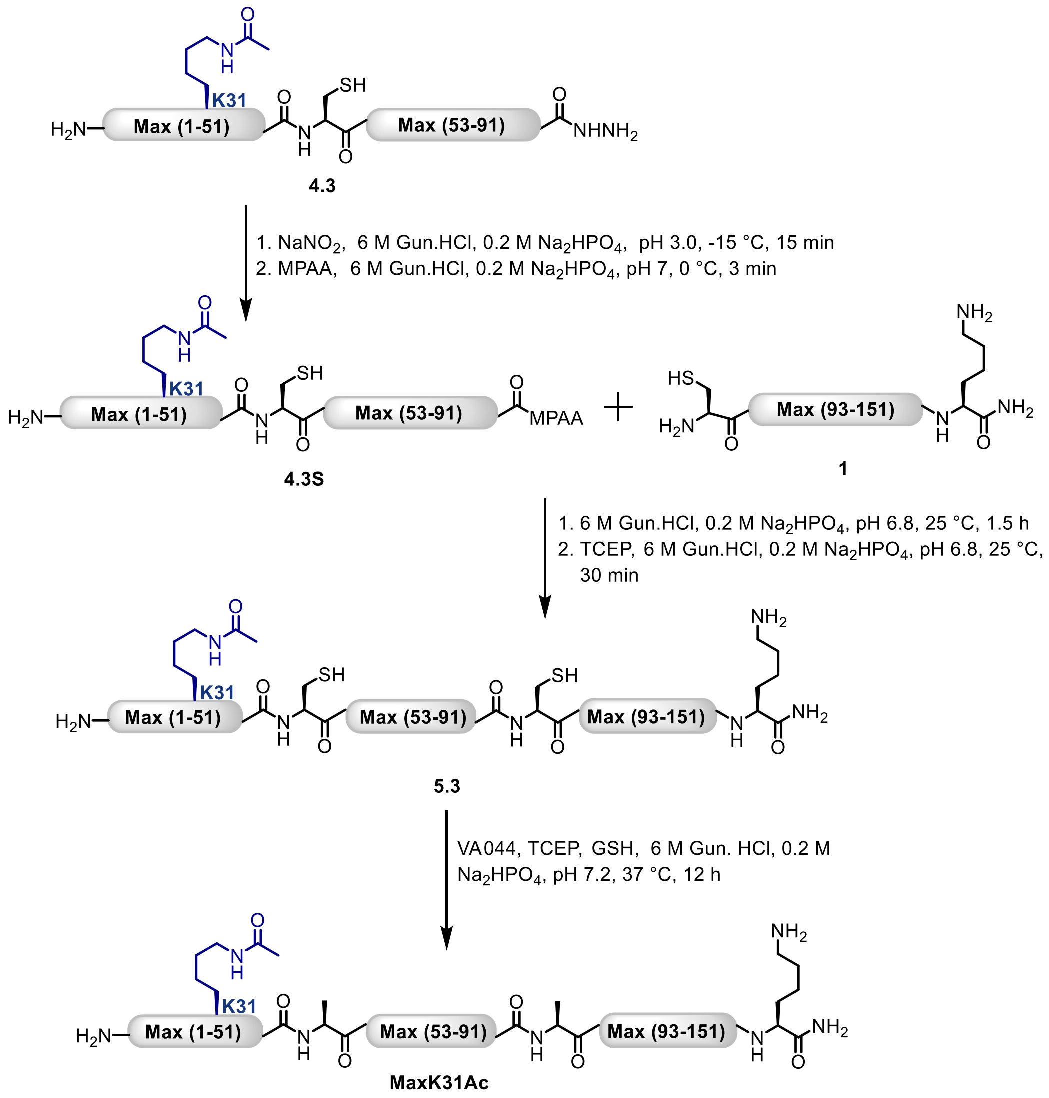


MaxK31Ac(1-91)-$NHNH_2$ **4.3** (4.6 mg, 1.4 equiv., 0.42 µmol, 5.6 mM) was dissolved in a 6 M Gun.HCl, 0.2 M $Na_2HPO_4$ buffer (75 µL) at pH 3.0 and cooled down to -15 °C by placing in an ice/salt bath. 5 µL of $NaNO_2$ (10 equiv., 4.2 µmol, 0.84 M; based on **4.3**) dissolved in water was added to the reaction mixture and allowed to react for 15 min at -15 °C with gentle mixing in repeated intervals. After 15 min 75 µL of MPAA (50 equiv., 21 µmol, 0.28 M; based on **4.3**) in 6 M Gun.HCl, 0.2 M $Na_2HPO_4$ buffer at pH 7 was added to the mixture and gently mixed for two-three minutes. The segment **1** Cys-Max(93-151) (2 mg, 1 equiv., 0.3 µmol, 2 mM) was then dissolved in the reaction mixture and the pH was adjusted to 6.8 using 1 *N* NaOH at 0 °C. The mixture was then incubated for 1.5 h at 25 °C and then 75 µL of TCEP (40 equiv., 16.8 µmol, 0.22 M; based on **4.3**) in 6 M Gun.HCl, 0.2 M $Na_2HPO_4$ buffer at pH 6.8 was added and continued incubating for 30 min at 25 °C. The reaction was monitored using analytical HPLC-MS (Method A described in Section 1.2). The ligation was completed in 2 h. After completion of the reaction, the crude

reaction was desalted by pipetting the reaction mixture into a 10 kDa molecular weight cutoff spin filter (Amicon® Ultra- 2mL, 10K). The reaction mixture was diluted with a 6 M Gun.HCl, 0.2 M $Na_2HPO_4$ buffer (pH 7.2) to 2.0 mL and concentrated to 1.0 mL by Centrifuging the spin filter at 5000 rpm for 15 min. This process was repeated four more times. In the final process, the reaction mixture was concentrated into 400 µL. After that, the reaction mixture was collected by reverse centrifuge and then treated with VA044 (80 µmol, 200 mM), TCEP (0.1 mmol, 250 mM), and L-Glutathione (GSH, 24 µmol, 60 mM) for 12 h. The progress of the reaction was monitored by analytical HPLC-MS using Method A (Section 1.2). After the completion of the reaction, purification was carried out using RP-HPLC (Method E described in Section 1.3) affording 1.7 mg (0.1 µmol) of final product **MaxK31Ac** as a white powder (33% yield, based on the limiting segment **1**).

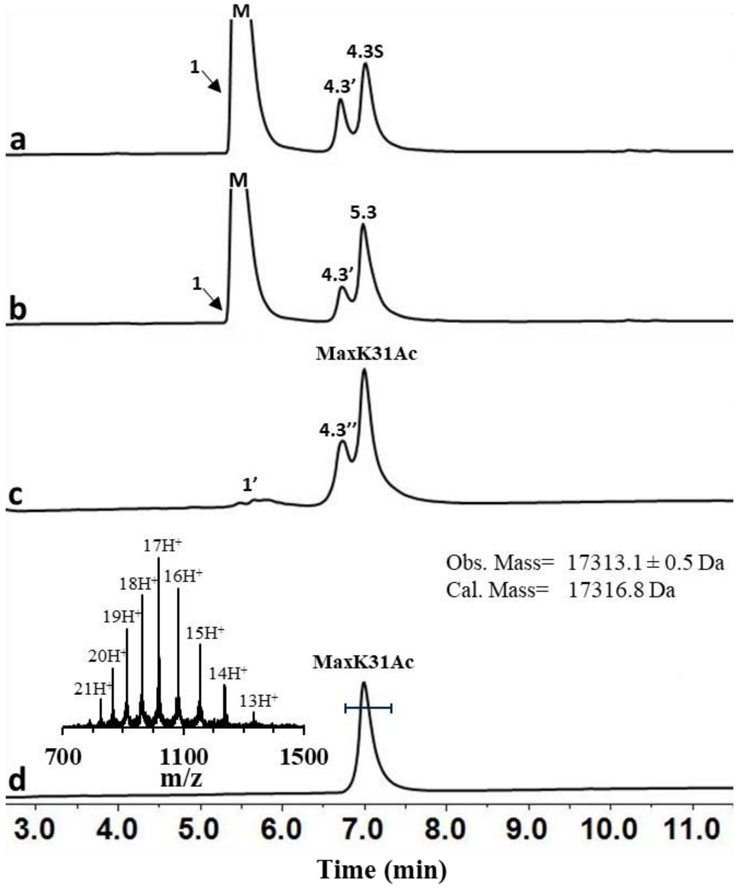


**Figure S10.** Analytical HPLC-MS of the progress of the native chemical ligation and one-pot desulfurization. **(a)** Ligation at t = 0 min; MaxK31Ac(1-91) thioester segment (**4.3S**), hydrolysis of MaxK31Ac(1-91) thioester (**4.3’**), Cys-Max(93-151) segment (**1**), and M=MPAA. **(b)** Crude ligation reaction at t = 120 min; ligated product **5.3** **(c)** Crude desulfurization reaction at t = 12 h; desulfurized segment **1** (**1’**), desulfurized-**4.3’** (**4.3’’**) **(d)** RP-HPLC purified **MaxK31Ac** with the observed mass 17313.1 ± 0.5 Da, calculated mass 17316.8 Da (average isotopes). The UV absorbance was monitored at 214 nm and the m/z data acquired over the marked region in the chromatogram.

## 5.6 Chemical synthesis of MaxK31AcK57Ac via native chemical ligation-desulfurization method

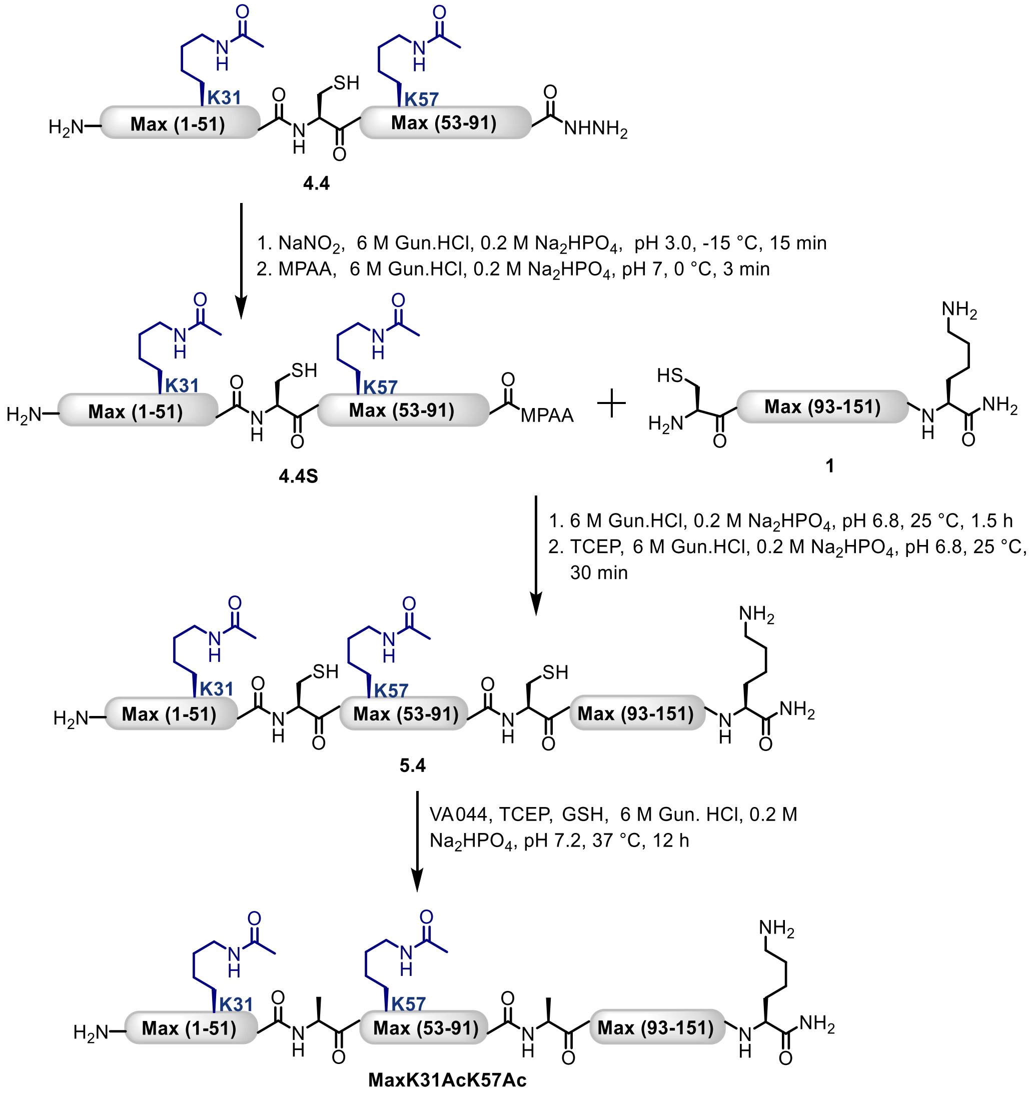


MaxK31AcK57Ac(1-91)-$NHNH_2$ **4.4** (4.6 mg, 1.4 equiv., 0.42 µmol, 5.6 mM) was dissolved in a 6 M Gun.HCl, 0.2 M $Na_2HPO_4$ buffer (75 µL) at pH 3.0 and cooled down to -15 °C by placing in an ice/salt bath. 5 µL of $NaNO_2$ (10 equiv., 4.2 µmol, 0.84 M; based on **4.4**) dissolved in water was added to the reaction mixture and allowed to react for 15 min at -15 °C with gentle mixing in repeated intervals. After 15 min 75 µL of MPAA (50 equiv., 21 µmol, 0.28 M; based on **4.4**) in 6 M Gun.HCl, 0.2 M $Na_2HPO_4$ buffer at pH 7 was added to the mixture and gently mixed for two-three minutes. The segment **1** Cys-Max(93-151) (2 mg, 1 equiv., 0.3 µmol, 2 mM) was then dissolved in the reaction mixture and the pH was adjusted to 6.8 using 1 *N* NaOH at 0 °C. The mixture was then incubated for 1.5 h at 25 °C and then 75 µL of TCEP (40 equiv., 16.8 µmol, 0.22 M; based on **4.4**) in 6 M Gun.HCl, 0.2 M $Na_2HPO_4$ buffer at pH 6.8 was added and continued incubating for 30 min at 25 °C. The reaction was monitored using analytical

HPLC-MS (Method A described in Section 1.2). The ligation was completed in 2 h. After completion of the reaction, the crude reaction was desalted by pipetting the reaction mixture into a 10 kDa molecular weight cutoff spin filter (Amicon® Ultra- 2mL, 10K). The reaction mixture was diluted with a 6 M Gun.HCl, 0.2 M $Na_2HPO_4$ buffer (pH 7.2) to 2.0 mL and concentrated to 1.0 mL by Centrifuging the spin filter at 5000 rpm for 15 min. This process was repeated four more times. In the final process, the reaction mixture was concentrated into 400 µL. After that, the reaction mixture was collected by reverse centrifuge and then treated with VA044 (80 µmol, 200 mM), TCEP (0.1 mmol, 250 mM), and L-Glutathione (GSH, 24 µmol, 60 mM) for 12 h. Progress of the reaction was monitored by analytical HPLC-MS using Method A (Section 1.2). After the completion of the reaction, purification was carried out using RP-HPLC (Method E described in Section 1.3) affording 1.9 mg (0.11 µmol) of final product **MaxK31AcK57Ac** as a white powder (37% yield, based on the limiting segment **1**).

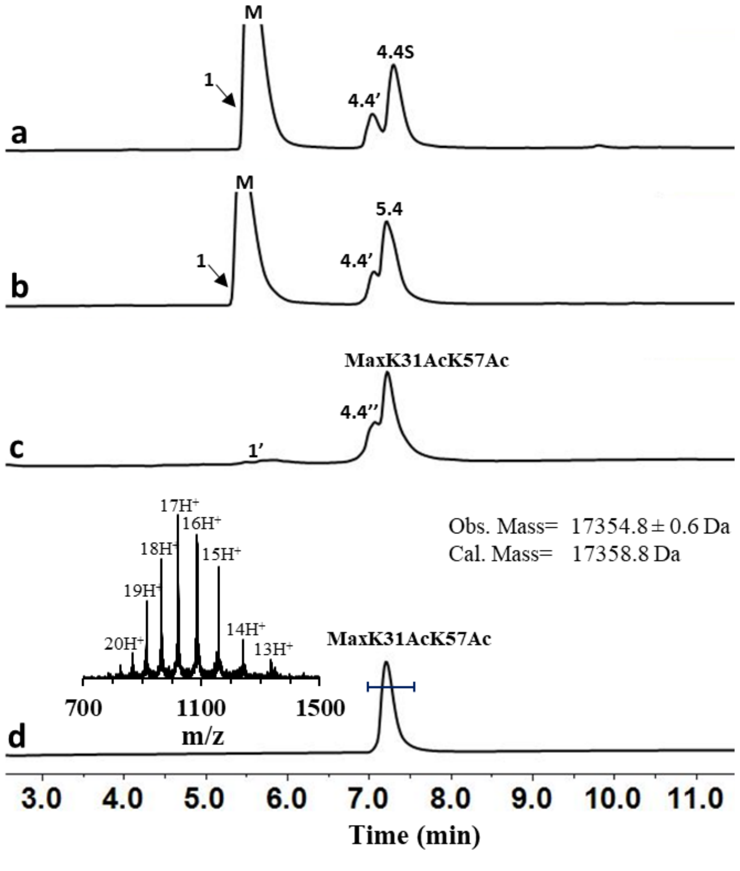


**Figure S11.** Analytical HPLC-MS of the progress of the native chemical ligation and one-pot desulfurization. **(a)** Ligation at t = 0 min; MaxK31AcK57Ac(1-91) thioester segment (**4.4S**), hydrolysis of MaxK31AcK57Ac(1-91) thioester (**4.4'**), Cys-Max(93-151) segment (**1**), and M=MPAA. **(b)** Crude ligation reaction at t = 120 min; ligated product **5.4** **(c)** Crude desulfurization reaction at t = 12 h; desulfurized segment **1** (**1'**), desulfurized-**4.4'** (**4.4''**) **(d)** RP-HPLC purified **MaxK31AcK57Ac** with the observed mass 17354.8 ± 0.6 Da, calculated mass 17358.8 Da (average isotopes). The UV absorbance was monitored at 214 nm and the m/z data acquired over the marked region in the chromatogram.

## 5.7 Chemical synthesis of T-MaxK57Ac via native chemical ligation-desulfurization method

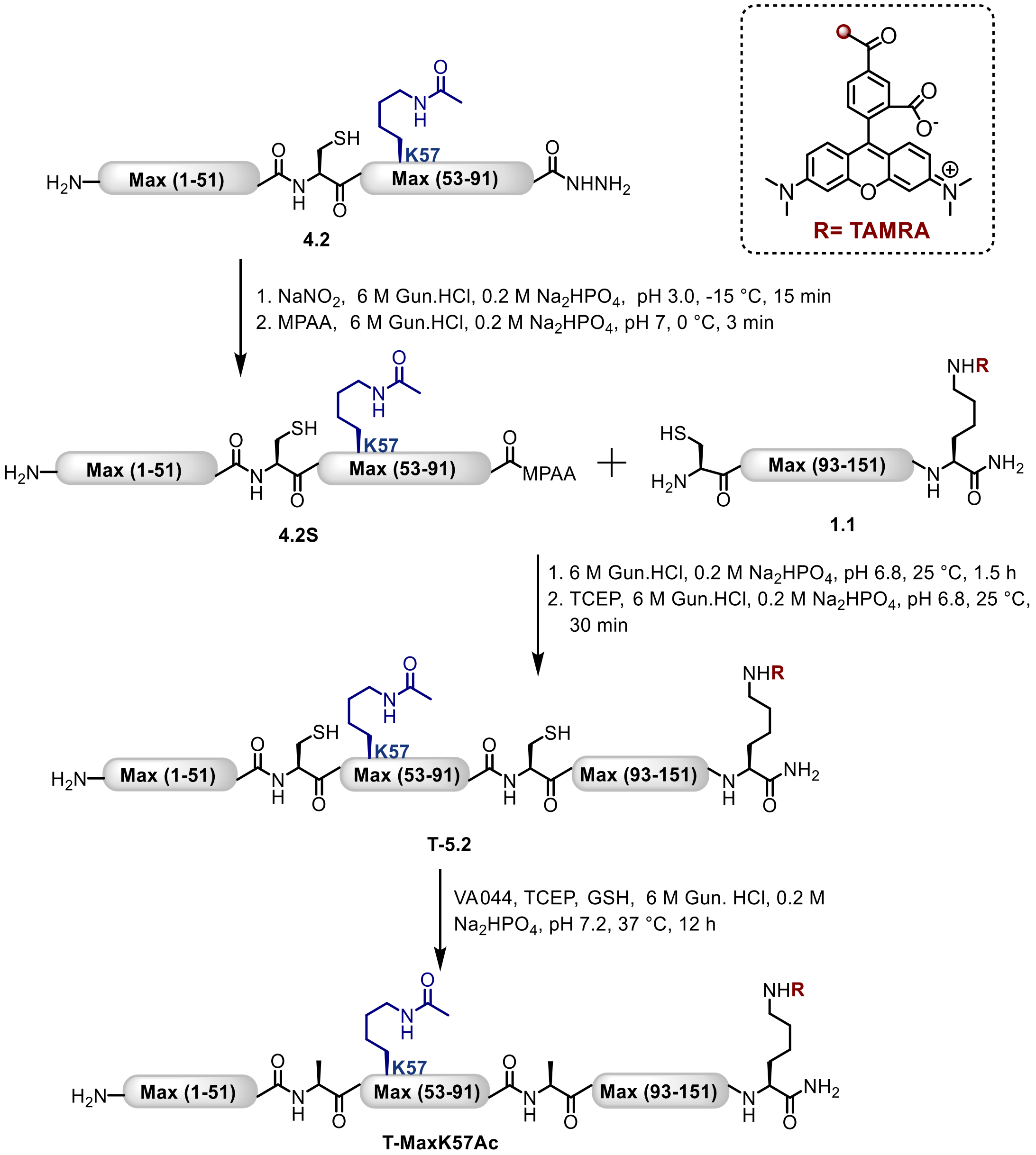


MaxK57Ac(1-91)-$NHNH_2$ **4.2** (4.4 mg, 1.4 equiv., 0.41 µmol, 5.4 mM) was dissolved in a 6 M Gun.HCl, 0.2 M $Na_2HPO_4$ buffer (75 µL) at pH 3.0 and cooled down to -15 °C by placing in an ice/salt bath. 5 µL of $NaNO_2$ (10 equiv., 4.1 µmol, 0.82 M; based on **4.2**) dissolved in water was added to the reaction mixture and allowed to react for 15 min at -15 °C with gentle mixing in repeated intervals. After 15 min 75 µL of MPAA (50 equiv., 20.5 µmol, 0.27 M; based on **4.2**) in 6 M Gun.HCl, 0.2 M $Na_2HPO_4$ buffer at pH 7 was added to the mixture and gently mixed for two-three minutes. The segment **1.1** Cys-Max(93-151)-TAMRA (2 mg, 1 equiv., 0.29 µmol, 1.8 mM) was then dissolved in the reaction mixture and the pH was adjusted to 6.8 using 1 *N* NaOH at 0 °C. The mixture was then incubated for 1.5 h at 25 °C and then 75 µL of TCEP

(40 equiv., 16.4 µmol, 0.22 M; based on **4.2**) in 6 M Gun.HCl, 0.2 M $Na_2HPO_4$ buffer at pH 6.8 was added and continued incubating for 30 min at 25 °C. The reaction was monitored using analytical HPLC-MS (Method A described in Section 1.2). The ligation was completed in 2 h. After completion of the reaction, the crude reaction was desalted by pipetting the reaction mixture into a 10 kDa molecular weight cutoff spin filter (Amicon® Ultra- 2mL, 10K). The reaction mixture was diluted with a 6 M Gun.HCl, 0.2 M $Na_2HPO_4$ buffer (pH 7.2) to 2.0 mL and concentrated to 1.0 mL by Centrifuging the spin filter at 5000 rpm for 15 min. This process was repeated four more times. In the final process, the reaction mixture was concentrated into 400 µL. After that, the reaction mixture was collected by reverse centrifuge and then treated with VA044 (80 µmol, 200 mM), TCEP (0.1 mmol, 250 mM), and L-Glutathione (GSH, 24 µmol, 60 mM) for 12 h. Progress of the reaction was monitored by analytical HPLC-MS using Method A (Section 1.2). After the completion of the reaction, purification was carried out using RP-HPLC (Method E described in Section 1.3) affording 1.3 mg (0.073 µmol) of final product **T-MaxK57Ac** as a red powder (25% yield, based on the limiting segment **1.1**).

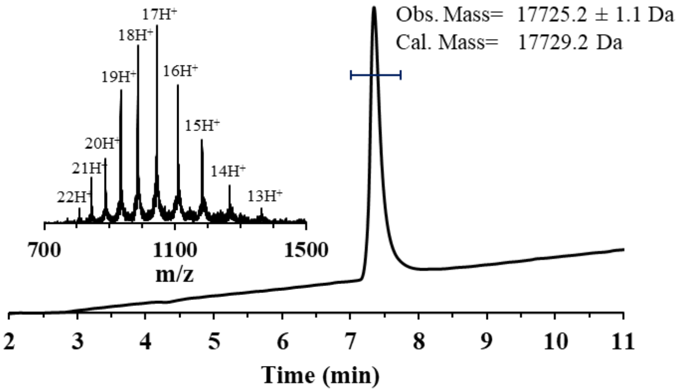


**Figure S12.** Analytical HPLC of purified **T-MaxK57Ac** and m/z spectrum with the observed mass 17725.2 ± 1.1 Da, calculated mass 17729.2 Da (average isotopes). The UV absorbance was monitored at 214 nm and the m/z data acquired over the marked region in the chromatogram. HPLC-MS analysis was carried out with Method A depicted in section 1.2 (5-50% acetonitrile gradient; 4.5% per min) and mass spectrometer.

## 5.8 Chemical synthesis of T-MaxK31Ac via native chemical ligation-desulfurization method

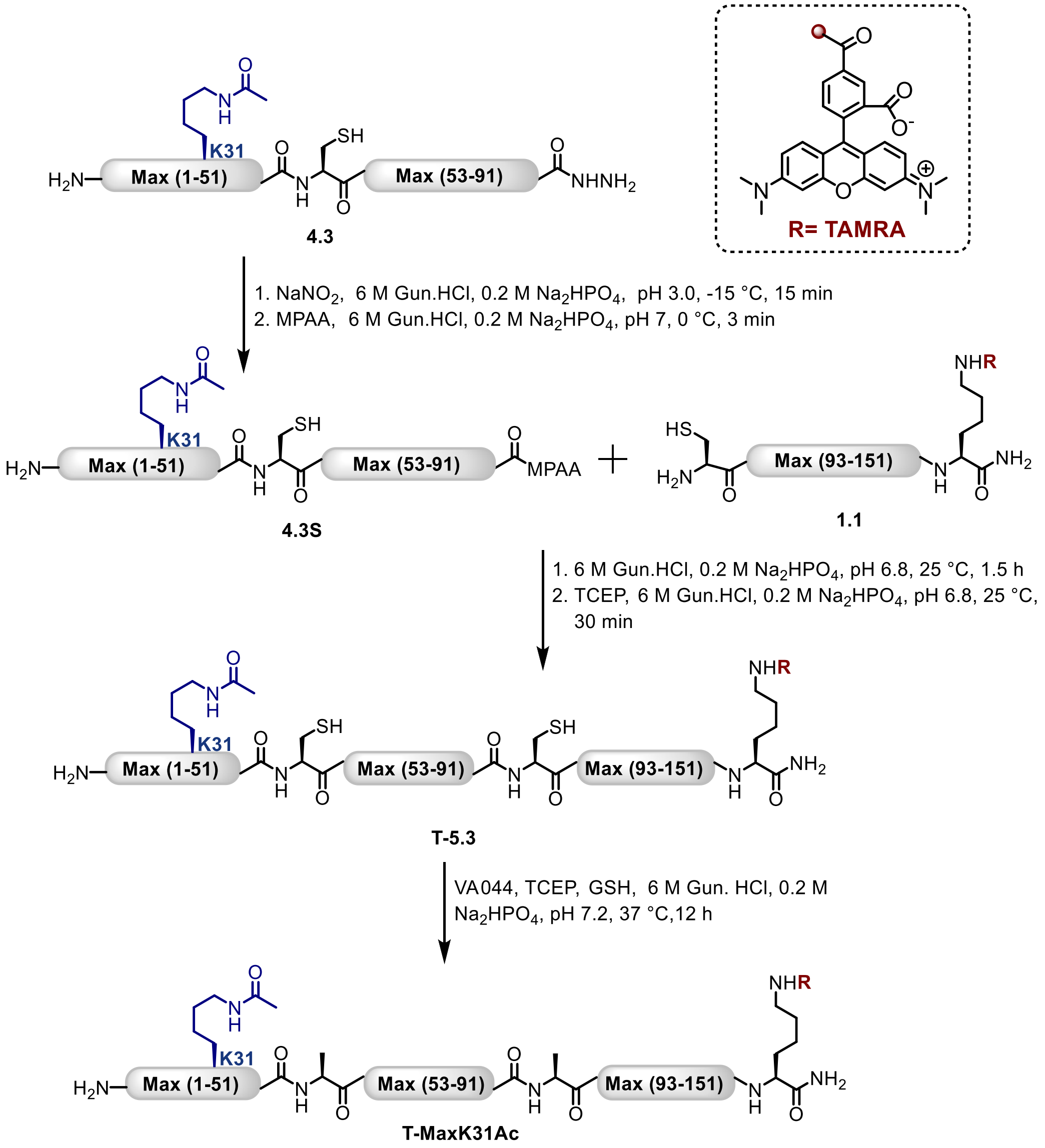


MaxK31Ac(1-91)-$NHNH_2$ **4.3** (4.4 mg, 1.4 equiv., 0.41 µmol, 5.4 mM) was dissolved in a 6 M Gun.HCl, 0.2 M $Na_2HPO_4$ buffer (75 µL) at pH 3.0 and cooled down to -15 °C by placing in an ice/salt bath. 5 µL of $NaNO_2$ (10 equiv., 4.1 µmol, 0.82 M; based on **4.3**) dissolved in water was added to the reaction mixture and allowed to react for 15 min at -15 °C with gentle mixing in repeated intervals. After 15 min 75 µL of MPAA (50 equiv., 20.5 µmol, 0.27 M; based on **4.3**) in 6 M Gun.HCl, 0.2 M $Na_2HPO_4$ buffer at pH 7 was added to the mixture and gently mixed for two-three minutes. The segment **1.1** Cys-Max(93-151)-TAMRA (2 mg, 1 equiv., 0.29 µmol, 1.8 mM) was then dissolved in the reaction mixture and the pH was adjusted to 6.8 using 1 *N* NaOH at 0 °C. The mixture was then incubated for 1.5 h at 25 °C and then 75 µL of TCEP (40 equiv., 16.4 µmol, 0.22 M; based on **4.3**) in 6 M Gun.HCl, 0.2 M $Na_2HPO_4$ buffer at pH 6.8 was added and continued incubating for 30 min at 25 °C. The reaction was monitored using analytical HPLC-MS

(Method A described in Section 1.2). The ligation was completed in 2 h. After completion of the reaction, the crude reaction was desalted by pipetting the reaction mixture into a 10 kDa molecular weight cutoff spin filter (Amicon® Ultra- 2mL, 10K). The reaction mixture was diluted with a 6 M Gun.HCl, 0.2 M $Na_2HPO_4$ buffer (pH 7.2) to 2.0 mL and concentrated to 1.0 mL by Centrifuging the spin filter at 5000 rpm for 15 min. This process was repeated four more times. In the final process, the reaction mixture was concentrated into 400 µL. After that, the reaction mixture was collected by reverse centrifuge and then treated with VA044 (80 µmol, 200 mM), TCEP (0.1 mmol, 250 mM), and L-Glutathione (GSH, 24 µmol, 60 mM) for 12 h. Progress of the reaction was monitored by analytical HPLC-MS using Method A (Section 1.2). After the completion of the reaction, purification was carried out using RP-HPLC (Method E described in Section 1.3) affording 1.4 mg (0.08 µmol) of final product **T-MaxK31Ac** as a red powder (28% yield, based on the limiting segment **1.1**).

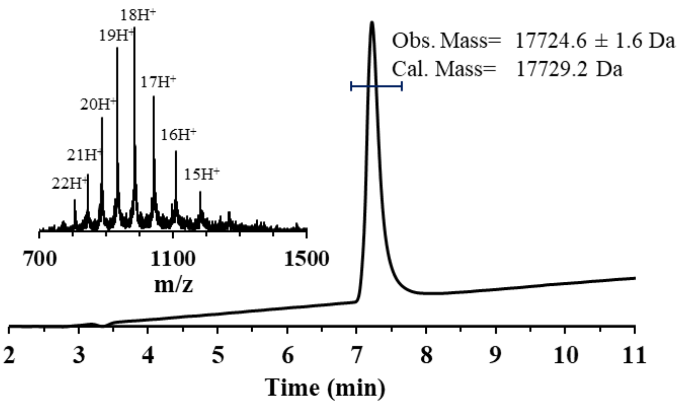


**Figure S13.** Analytical HPLC of purified **T-MaxK31Ac** and m/z spectrum with the observed mass 17724.6 ± 1.6 Da, calculated mass 17729.2 Da (average isotopes). The UV absorbance was monitored at 214 nm and the m/z data acquired over the marked region in the chromatogram. HPLC-MS analysis was carried out with Method A depicted in section 1.2 (5-50% acetonitrile gradient; 4.5% per min) and mass spectrometer.

## 5.9 Chemical synthesis of T-MaxK31AcK57Ac via native chemical ligation-desulfurization method

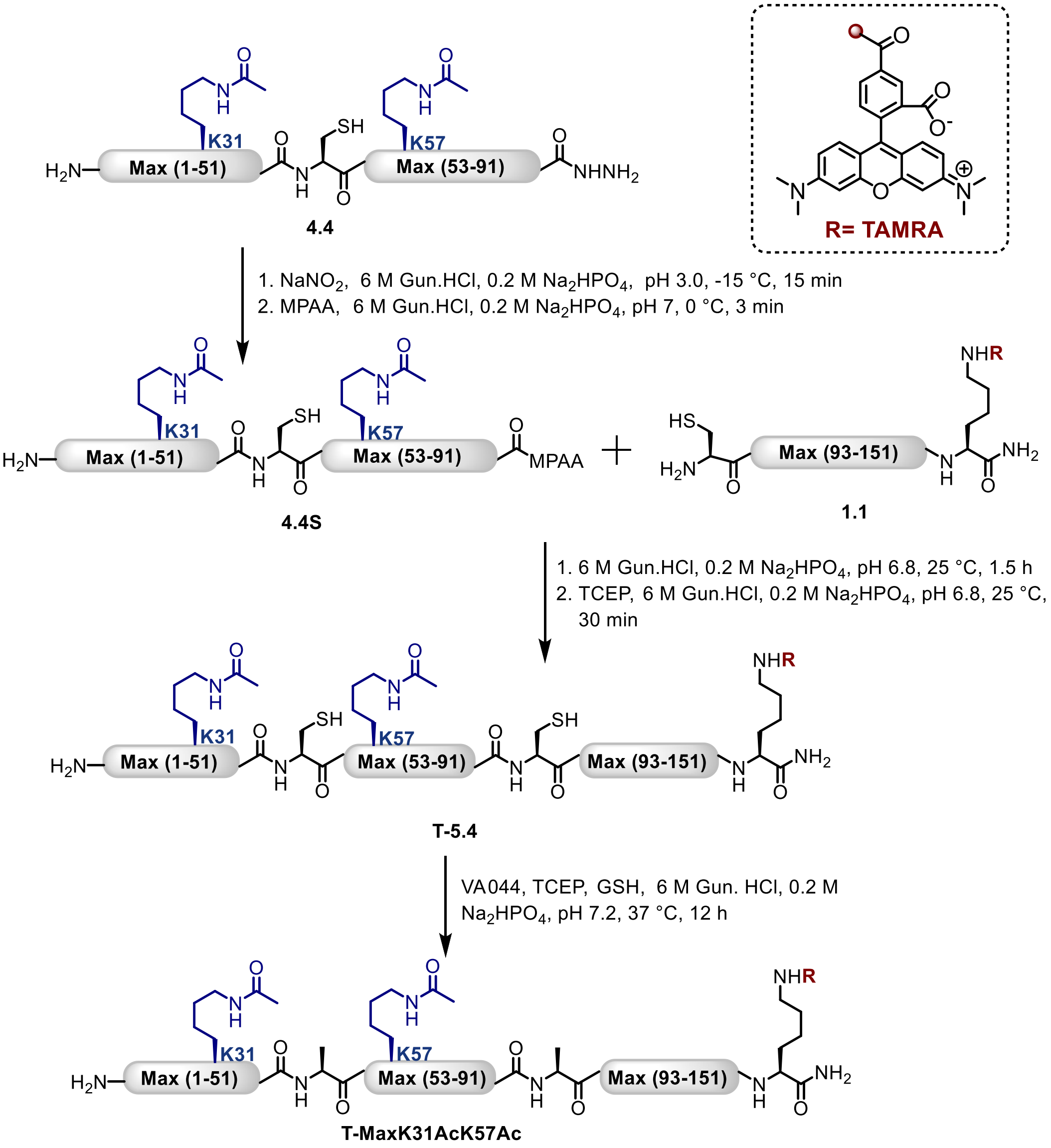


MaxK31AcK57Ac(1-91)-NHNH$_2$ **4.4** (4.5 mg, 1.4 equiv., 0.41 µmol, 5.5 mM) was dissolved in a 6 M Gun.HCl, 0.2 M $Na_2HPO_4$ buffer (75 µL) at pH 3.0 and cooled down to -15 °C by placing in an ice/salt bath. 5 µL of $NaNO_2$ (10 equiv., 4.1 µmol, 0.82 M; based on **4.4**) dissolved in water was added to the reaction mixture and allowed to react for 15 min at -15 °C with gentle mixing in repeated intervals. After 15 min 75 µL of MPAA (50 equiv., 20.5 µmol, 0.27 M; based on **4.4**) in 6 M Gun.HCl, 0.2 M $Na_2HPO_4$ buffer at pH 7 was added to the mixture and gently mixed for two-three minutes. The segment **1.1** Cys-Max(93-151)-TAMRA (2 mg, 1 equiv., 0.29 µmol, 1.8 mM) was then dissolved in the reaction mixture and the pH was adjusted to 6.8 using 1 *N* NaOH at 0 °C. The mixture was then incubated for 1.5 h at 25 °C and then 75 µL of TCEP (40 equiv., 16.4 µmol, 0.22 M; based on **4.4**) in 6 M Gun.HCl, 0.2 M $Na_2HPO_4$ buffer

at pH 6.8 was added and continued incubating for 30 min at 25 °C. The reaction was monitored using analytical HPLC-MS (Method A described in Section 1.2). The ligation was completed in 2 h. After completion of the reaction, the crude reaction was desalted by pipetting the reaction mixture into a 10 kDa molecular weight cutoff spin filter (Amicon® Ultra- 2mL, 10K). The reaction mixture was diluted with a 6 M Gun.HCl, 0.2 M $Na_2HPO_4$ buffer (pH 7.2) to 2.0 mL and concentrated to 1.0 mL by Centrifuging the spin filter at 5000 rpm for 15 min. This process was repeated four more times. In the final process, the reaction mixture was concentrated into 400 µL. After that, the reaction mixture was collected by reverse centrifuge and then treated with VA044 (80 µmol, 200 mM), TCEP (0.1 mmol, 250 mM), and L-Glutathione (GSH, 24 µmol, 60 mM) for 12 h. Progress of the reaction was monitored by analytical HPLC-MS using Method A (Section 1.2). After the completion of the reaction, purification was carried out using RP-HPLC (Method E described in Section 1.3) affording 1.6 mg (0.09 µmol) of final product **T-MaxK31AcK57Ac** as a red powder (31% yield, based on the limiting segment **1.1**).

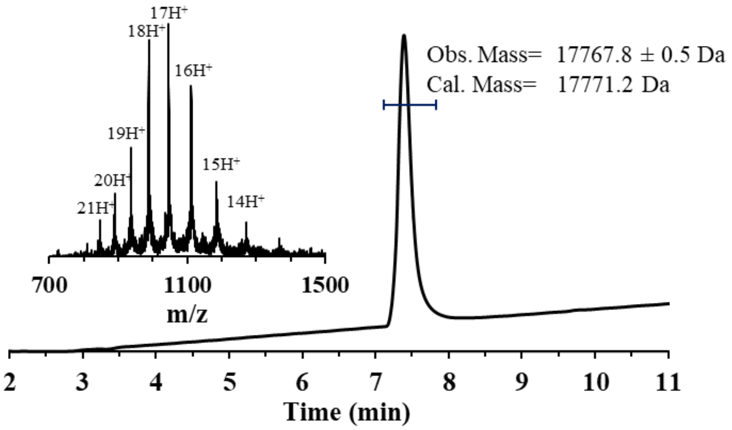


**Figure S14.** Analytical HPLC of purified **T-MaxK31AcK57Ac** and m/z spectrum with the observed mass 17767.8 ± 0.5 Da, calculated mass 17771.2 Da (average isotopes). The UV absorbance was monitored at 214 nm and the m/z data acquired over the marked region in the chromatogram. HPLC-MS analysis was carried out with Method A depicted in section 1.2 (5-50% acetonitrile gradient; 4.5% per min) and mass spectrometer.

## 5.10 Chemical synthesis of wt-Max and T-wt-Max

The chemical synthesis of **wt-Max** and **T-wt-Max** was carried out according to the previous literature.[6] The analytical HPLC-MS spectrum of the final products are shown below.

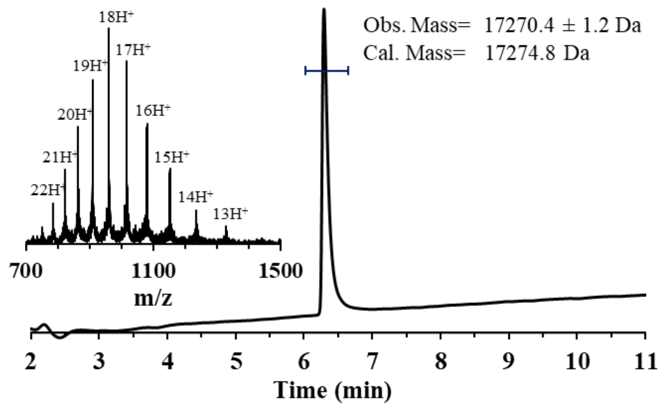


**Figure S15.** Analytical HPLC of purified **wt-Max** and m/z spectrum with the observed mass 17270.4 ± 1.2 Da, calculated mass 17274.8 Da (average isotopes). The UV absorbance was monitored at 214 nm and the m/z data acquired over the marked region in the chromatogram. HPLC-MS analysis was carried out with Method A depicted in section 1.2 (5-50% acetonitrile gradient; 4.5% per min) and mass spectrometer.

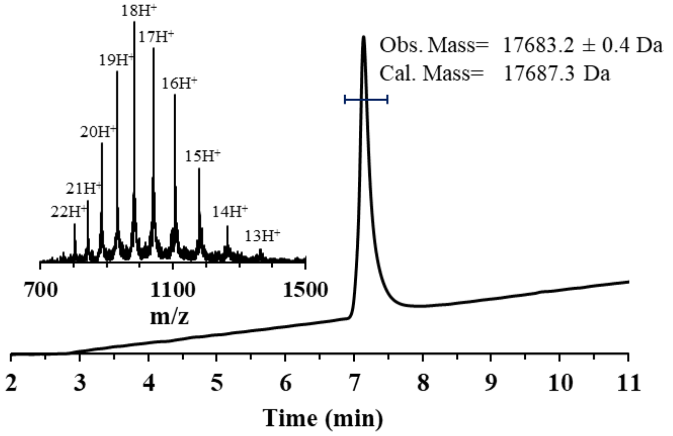


**Figure S16.** Analytical HPLC of purified **T-wt-Max** and m/z spectrum with the observed mass 17683.2 ± 0.4 Da, calculated mass 17687.3 Da (average isotopes). The UV absorbance was monitored at 214 nm and the m/z data acquired over the marked region in the chromatogram. HPLC-MS analysis was carried out with Method A depicted in section 1.2 (5-50% acetonitrile gradient; 4.5% per min) and mass spectrometer.

# 6. Folding and analysis of Max variants

Synthetic Max variants were dissolved in DMSO (2 mM) and then diluted into 10 mM MES, 150 mM KCl, 1 mM $MgCl_2$, 10% glycerol buffer (pH 6) using an Amicon® Ultra- 0.5 mL 3K MWCO spin filtration unit to provide the desired Max variants in 50 μM final concentration in 10 mM MES, 150 mM KCl, 1 mM $MgCl_2$, 10% glycerol buffer (pH 6). The concentrations of Max analogs were determined using a NanoDrop ND-1000 spectrophotometer.

The folded Max analogs were characterized via Size Exclusion Chromatography (SEC) using ÄKTA Pure on a Superdex 75 column. The column was equilibrated with a buffer consisting of 10 mM MES, 500 mM KCl, 100 mM NaCl and 1 mM $MgCl_2$. All Max variants were eluted as a single peak at around 9 ml, which corresponds to the mass of a Max homodimer (~35 kDa).

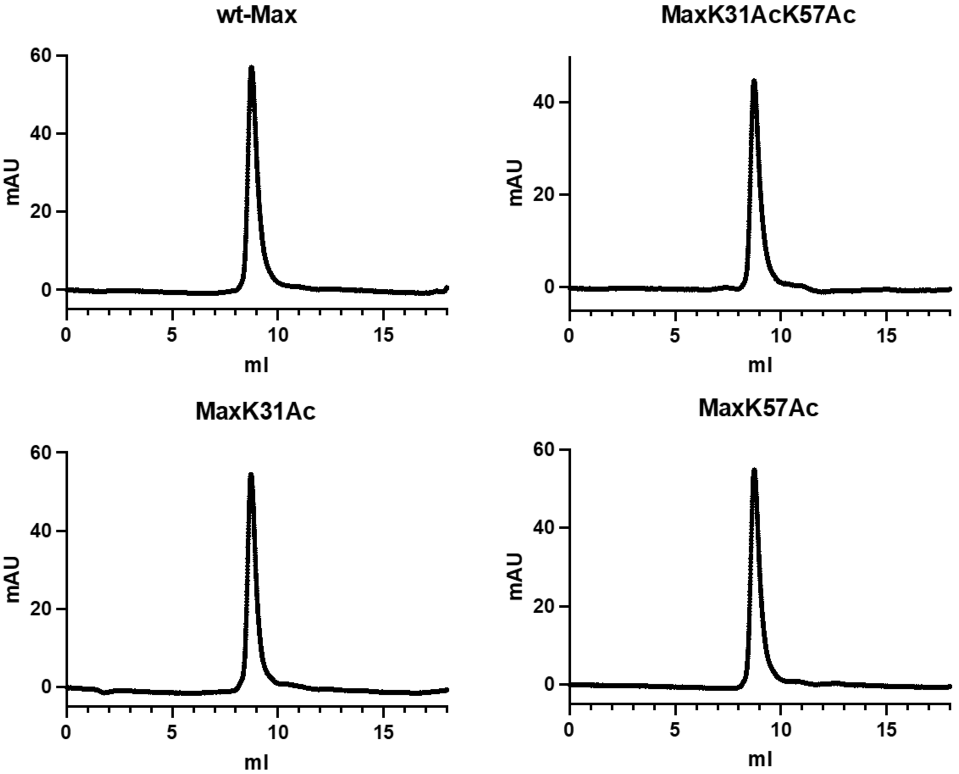


**Figure S17.** The size exclusion chromatography (SEC) experiments show all Max variants are homogeneous species with a molecular weight corresponding to Max homodimers (35 kDa). The UV absorbance was monitored at 220 nm.

## 7. Circular Dichroism (CD) Analysis

CD analysis was carried out using Chirascan circular dichroism spectrometer with a 0.1 mm path length quartz cuvette. 10 µM, 50.0 µL protein was prepared in 10 mM MES, 150 mM KCl, 1 mM $MgCl_2$, 10% glycerol buffer (pH 6.0). CD spectra of all samples were recorded in triplicates at 20 °C from 180 nm to 270 nm in 1.0 nm step with 3.0 nm slit bandwidth and three seconds averaging times at each wavelength.

## 8. DNA-Binding Analysis and Electrophoretic Mobility-Shift Assay (EMSA)

An E-box DNA probe (2.0 µL, 10.0 µM) was added to a 0.6 mL Eppendorf tube containing either 16.0 µL or 14.0 µL of 10 mM MES buffer,150 mM KCl, 1 mM MgCl2, and 10% glycerol, pH 6. This was followed by the addition of the target protein analog, prepared in the same buffer, at volumes of 2.0 µL (20.0 µM) or 4.0 µL (20.0 µM). The final concentrations in the reaction mixture were 1.0 µM for DNA and either 2.0 µM or 4.0 µM for the protein. The tube was thoroughly mixed by pipetting up and down and then incubated at room temperature for 30 minutes. Concurrently, a 10% TBE gel (1.0 mm thick, 10 wells) was pre-run in 1x TBE buffer at 90V for 45 minutes. After incubation, the DNA-binding activity of each protein analog was evaluated by EMSA, which separates bound DNA-protein complexes (indicating interaction) from unbound DNA (free DNA). For this assay, 5.0 µL of the DNA-protein mixture was mixed with 1.0 µL of DNA Loading Dye (6X). This mixture was then loaded onto a pre-run polyacrylamide gel and electrophoresed at 90V for 60 minutes. Following electrophoresis, the gel was washed three times with water (30 seconds each) to remove excess ions and stained with Ethidium Bromide in 1x TBE buffer for 15 minutes at room temperature. Bands representing bound and unbound DNA were visualized using an A2S Vilber Fusion FX imager.

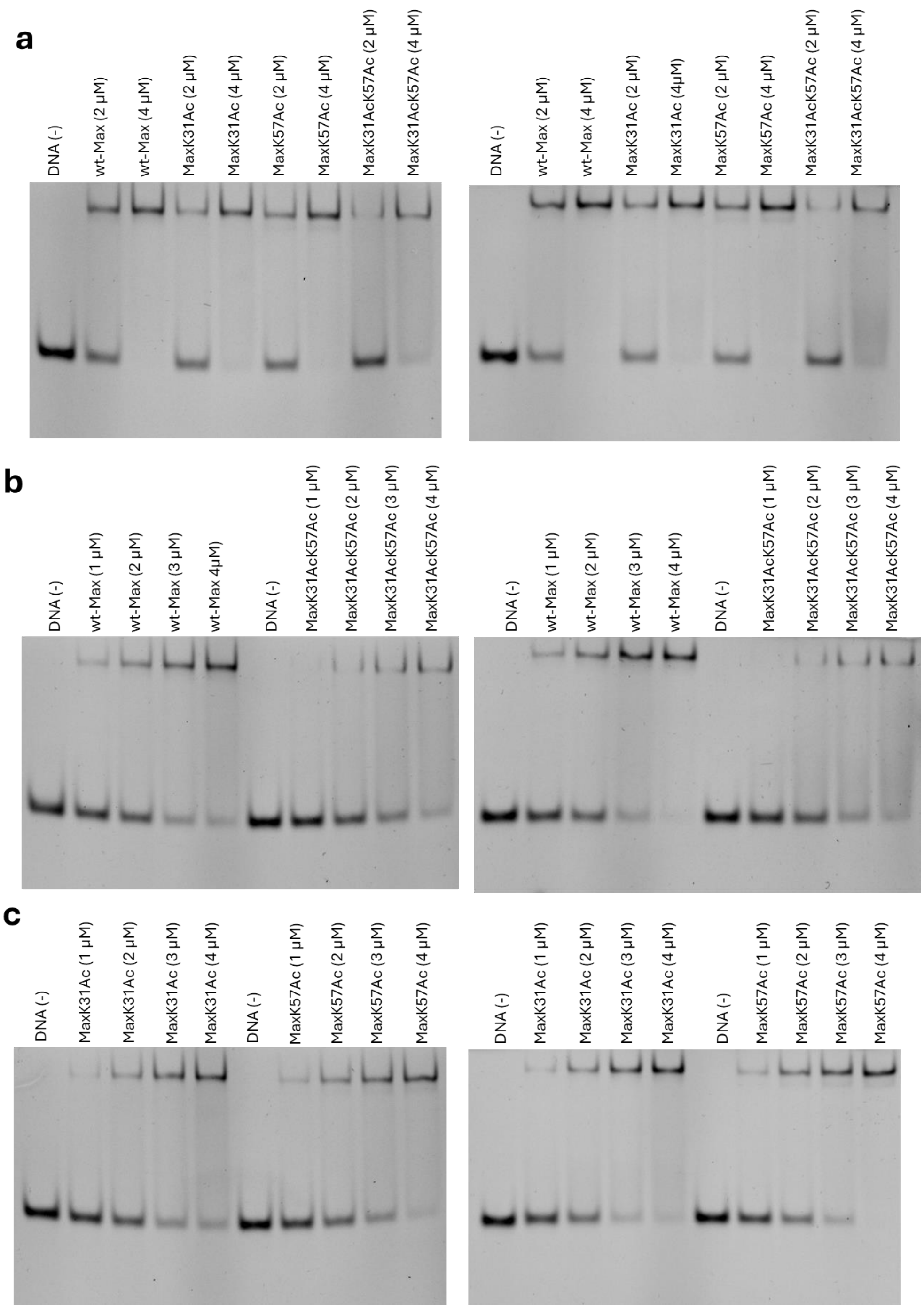
a
DNA (-)
wt-Max (2 μM)
wt-Max (4 μM)
MaxK31Ac (2 μM)
MaxK31Ac (4 μM)
MaxK57Ac (2 μM)
MaxK57Ac (4 μM)
MaxK31AcK57Ac (2 μM)
MaxK31AcK57Ac (4 μM)
DNA (-)
wt-Max (2 μM)
wt-Max (4 μM)
MaxK31Ac (2 μM)
MaxK31Ac (4μM)
MaxK57Ac (2 μM)
MaxK57Ac (4 μM)
MaxK31AcK57Ac (2 μM)
MaxK31AcK57Ac (4 μM)
b
DNA (-)
wt-Max (1 μM)
wt-Max (2 μM)
wt-Max (3 μM)
wt-Max 4μM)
DNA (-)
MaxK31AcK57Ac (1 μM)
MaxK31AcK57Ac (2 μM)
MaxK31AcK57Ac (3 μM)
MaxK31AcK57Ac (4 μM)
DNA (-)
wt-Max (1 μM)
wt-Max (2 μM)
wt-Max (3 μM)
wt-Max (4 μM)
DNA (-)
MaxK31AcK57Ac (1 μM)
MaxK31AcK57Ac (2 μM)
MaxK31AcK57Ac (3 μM)
MaxK31AcK57Ac (4 μM)
c
DNA (-)
MaxK31Ac (1 μM)
MaxK31Ac (2 μM)
MaxK31Ac (3 μM)
MaxK31Ac (4 μM)
DNA (-)
MaxK57Ac (1 μM)
MaxK57Ac (2 μM)
MaxK57Ac (3 μM)
MaxK57Ac (4 μM)
DNA (-)
MaxK31Ac (1 μM)
MaxK31Ac (2 μM)
MaxK31Ac (3 μM)
MaxK31Ac (4 μM)
DNA (-)
MaxK57Ac (1 μM)
MaxK57Ac (2 μM)
MaxK57Ac (3 μM)
MaxK57Ac (4 μM)

d

e

f

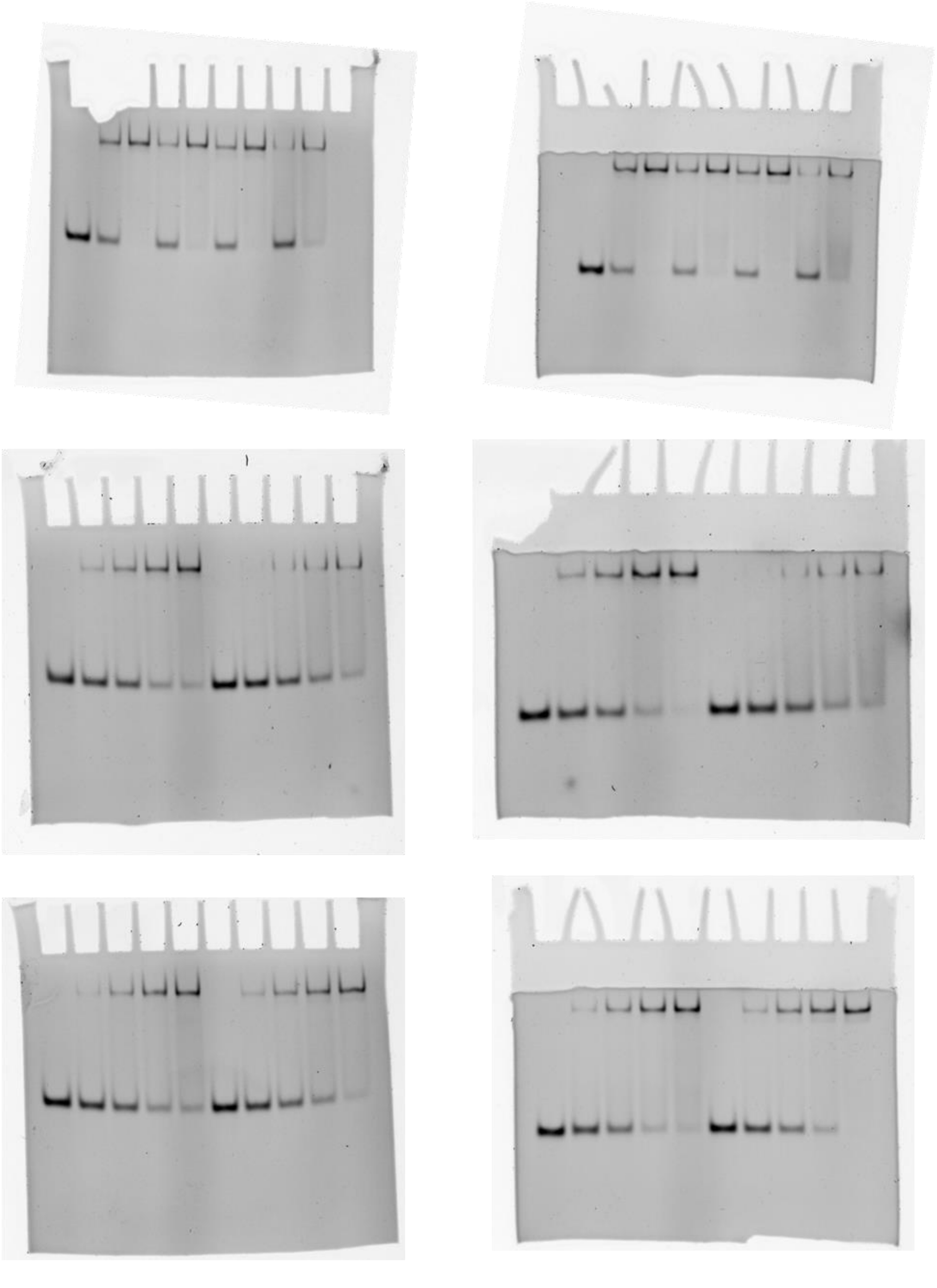

**Figure S18. (a)** EMSA experiment of all Max analogs along with replicate. Conditions: 1 μM DNA probe and 2 μM and 4 μM protein. **(b).** EMSA experiment of **wt-Max** and **MaxK31AcK57Ac** analogs along with replicate. Conditions: 1 μM DNA probe and 1 μM, 2 μM, 3 μM, and 4 μM protein. **(c).** EMSA experiment of **MaxK31Ac** and **MaxK57Ac** analogs along with replicate. Conditions: 1 μM DNA probe and 1 μM, 2 μM, 3 μM, and 4 μM protein. (**d-f**) are the original gels of a, b, and c respectively.

## 9. Octet BioLayer Interferometry Binding Assay (BLI)

Biolayer interferometry (BLI) assays were performed using an Octet Red R84 System (ForteBio; Menlo Park,CA) in 96 well plates. Streptavidin Octet biosensors (ForteBio; Menlo Park, CA) were dipped into 0.1% BSA, 0.02% Tween-20, 1x PBS (kinetic buffer) for 10 min and then 60 more seconds in the kinetic buffer to obtain the baseline. Then the sensors were dipped into 200 µL 130 nM of biotinylated E-box DNA probe in the kinetic buffer for the loading step (120 sec). Sensors were then dipped into the kinetic buffer for 60 sec. Next, the tips were loaded with Max prepared in kinetic buffer at the indicated concentrations for 150 sec to obtain the association curve. Finally, the tips were dipped into the kinetic buffer for 200 sec to obtain the dissociation curve. Measurements were carried out at 25 °C. Data were analyzed within the ForteBio Data Analysis software. The association and dissociation curves are fitted with Fortebio Biosystems (global fitting algorism) to obtain the $K_D$. Kinetic $K_D$ is reported.

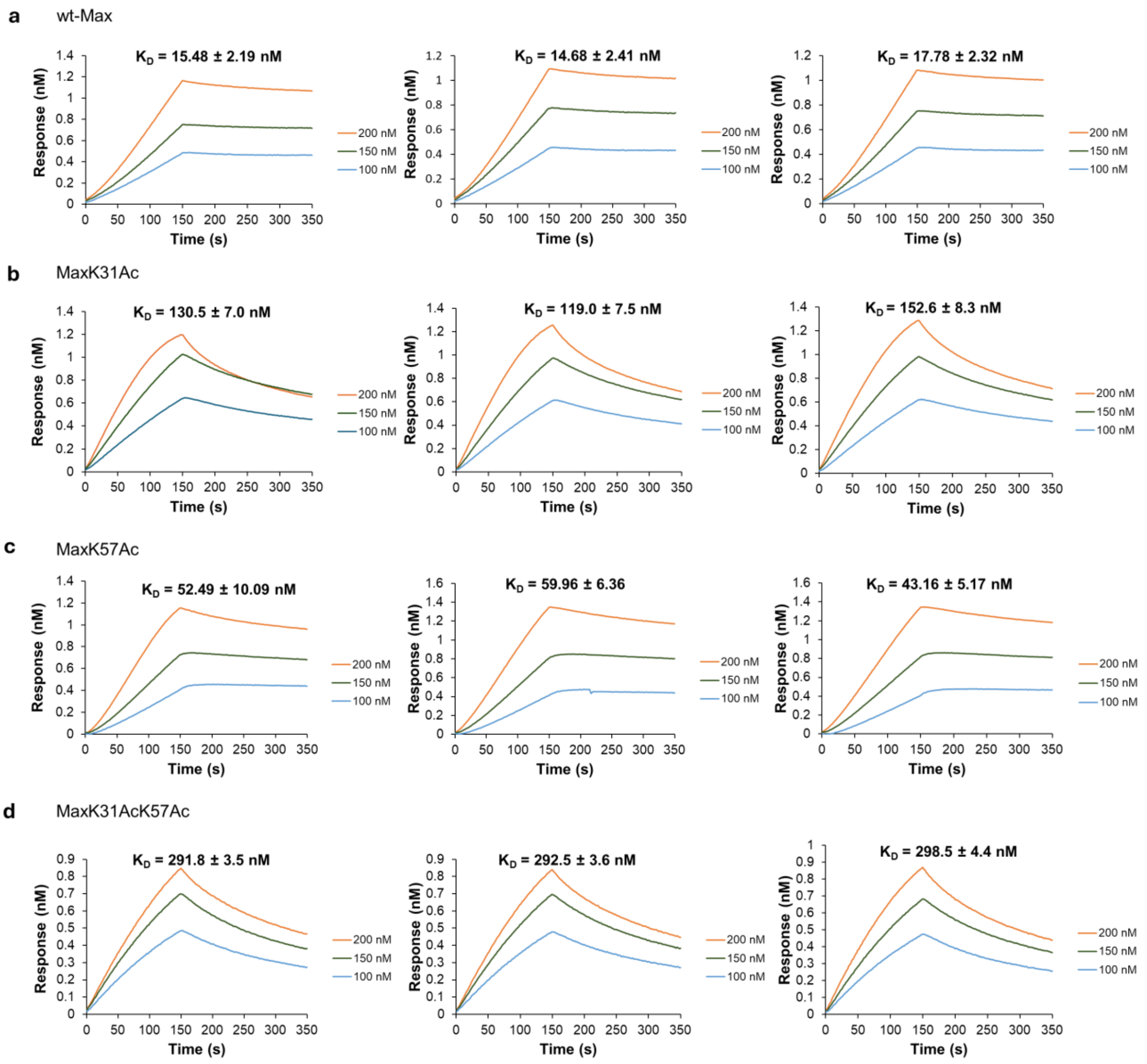


**Figure S19.** BLI analysis of Max variants with the E-box-1 DNA probe; the association and dissociation curves of proteins **wt-Max (a)**, **MaxK31Ac (b)**, **MaxK57Ac (c)**, and **MaxK31AcK57Ac (d)** with E-box-1 DNA probe along with the triplicates.

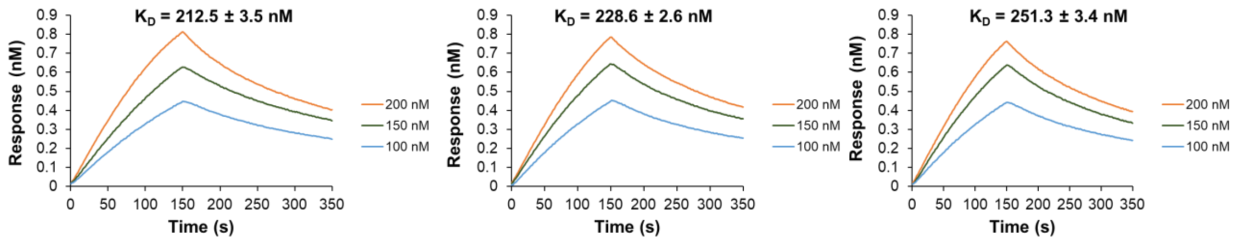


**Figure S20.** BLI analysis of **MaxK31AcK57Ac** with the E-box-2 DNA probe; the association and dissociation curves of protein **MaxK31AcK57Ac** with E-box-2 DNA probe along with the triplicates.

# 10. Absorption and Emission Spectra of TAMRA Labeled Max Analogs

The absorption and emission spectra were measured using a BioTek synergy H4 hybrid multi-well plate reader in clear flat-bottom polystyrene 384-well microplates (Greiner). The absorption spectra were measured over the range from 300 nm to 700 nm. The emission spectra were obtained by exciting the TAMRA-labelled analogues at 532 nm and 500 nm, respectively, and measured over the range from 552 nm/520 nm to 800 nm.

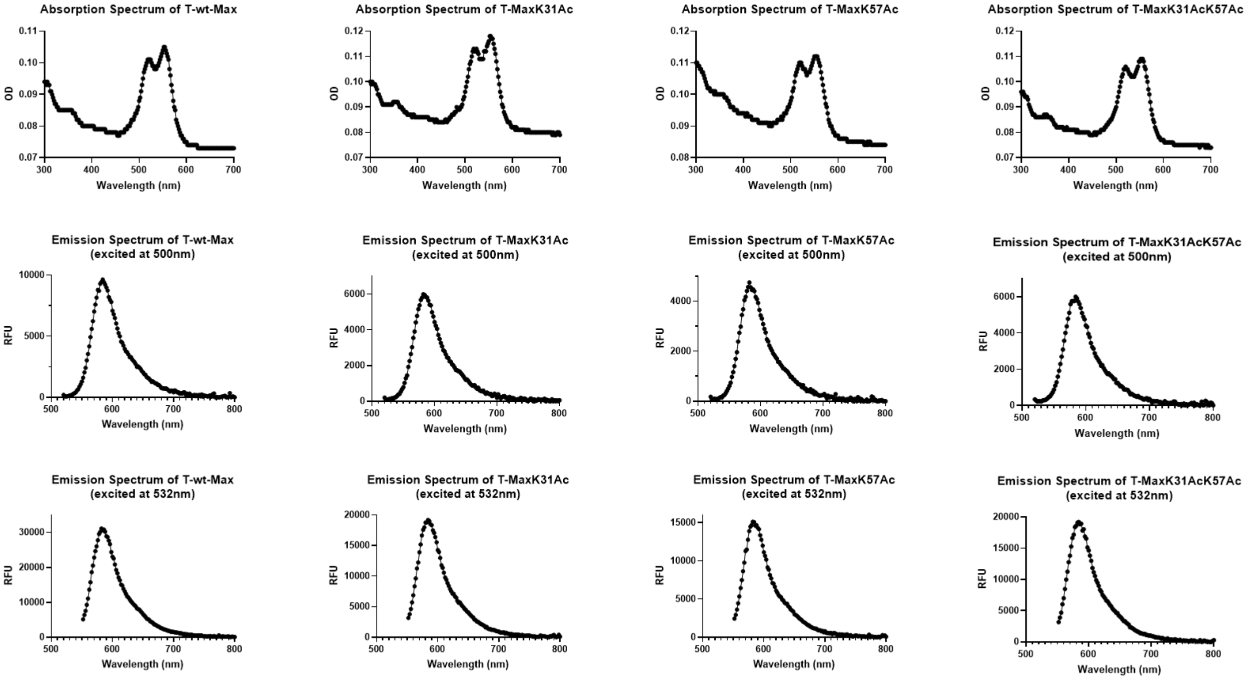


**Figure S21.** Characterization of the absorption and emission spectra for all TAMRA-labeled Max variants. All absorption spectra exhibit double maxima between 520nm and 580nm. In all emission spectra, RFU reaches maximum around 580nm.

## 11. Protein binding microarray analysis

PBM experiments were performed as described previously,[7,8] with a modification: we detected fluorescence directly from the TAMRA tag on the protein instead of using a fluorescently labeled antibody. This adaptation simplified the protocol by reducing the number of washing steps required. Universal PBM and custom-designed 4x180k microarray were used (Agilent Technologies; AmadID 087313). The universal PBM used in this study is the same as described previously,[8,9] containing all possible 10mer sequences. The custom-designed array contains DNA sequences with a Max DNA binding motif and various flanks. It contains six libraries of DNA sequences (which we refer to as libraries 1 to 6), all of which have a specific binding core flanked by various sequences. In library1, three core motifs with different binding affinity levels were chosen, and they were flanked by all possible 3mers on the left and right. In library2, we included all the single bp mutations on an extended 10-bp binding site, and each of them has all possible 2mers on its left and right. In library3, five 14-bp long core motif with different levels of binding affinities were included, each with all possible 2-mers on its left and right. We also added a Library that contains randomly selected genomic regions bound in vivo by c-Myc, Max, or Mad2 (ChIP-seq P < 10^(-10) in HeLaS3 or K562 cells which contain at least two consecutive 8-mers with universal PBM E-score > 0.4; GEO accession: GSE59845),[10,11] and a negative control group, and the sequences are the genomic unbound regions in vivo (GEO accession: GSE59845).[10,11] The full design can be found in Figure S18 and the full list of sequences 4x180k_MAX_sequences.txt.

The microarrays were double stranded by solid-phase primer extension with Thermo Sequenase DNA Polymerase (Cytiva) and regular dNTP. After blocking with 2% non-fat dry milk, microarrays were incubated with fluorescent Max proteins. The protein concentration was in the range of 50nM and 200nM; the protein mixture is PBS based, containing 2% non-fat dry milk, 200 ng/μL BSA, 50 ng/μL Salmon Testes DNA, and 0.02% TX-100. The fluorescence was scanned with a GenePix® 4400A scanner at 532nm at 2.5um resolution, and the fluorescence intensity was extracted with GenePix software for all probes in the array. Each sequence has 6 replicate probes, and all probes were randomized on the array. For each probe, we report the median pixel intensity, and for each sequence, we report the median probe intensity.

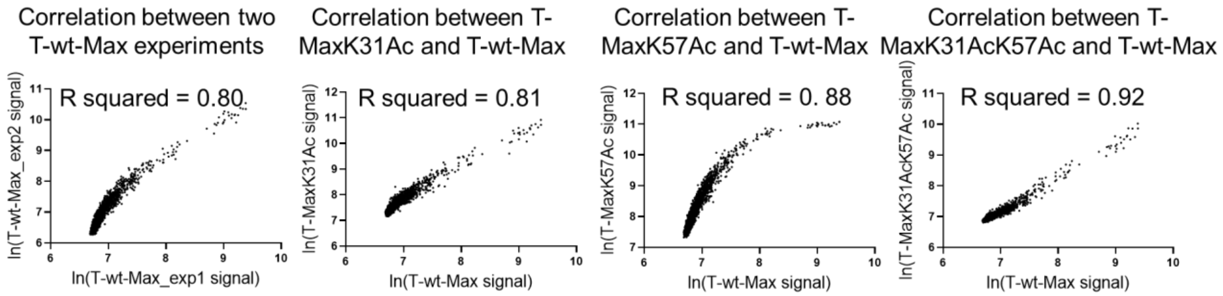


**Figure S22.** Consistent trends in 8-mer sequences across all variants. The correlation between the 8mer median intensity values of acetylated Max variants (**T-MaxK31Ac, T-MaxK57Ac** and **T-MaxK31AcK57Ac**) and **T-wt-Max** is similar to the correlation between two independent experiments performed for **T-wt-Max**. Demonstrating similar binding preferences toward 8-mer cores for all variants.

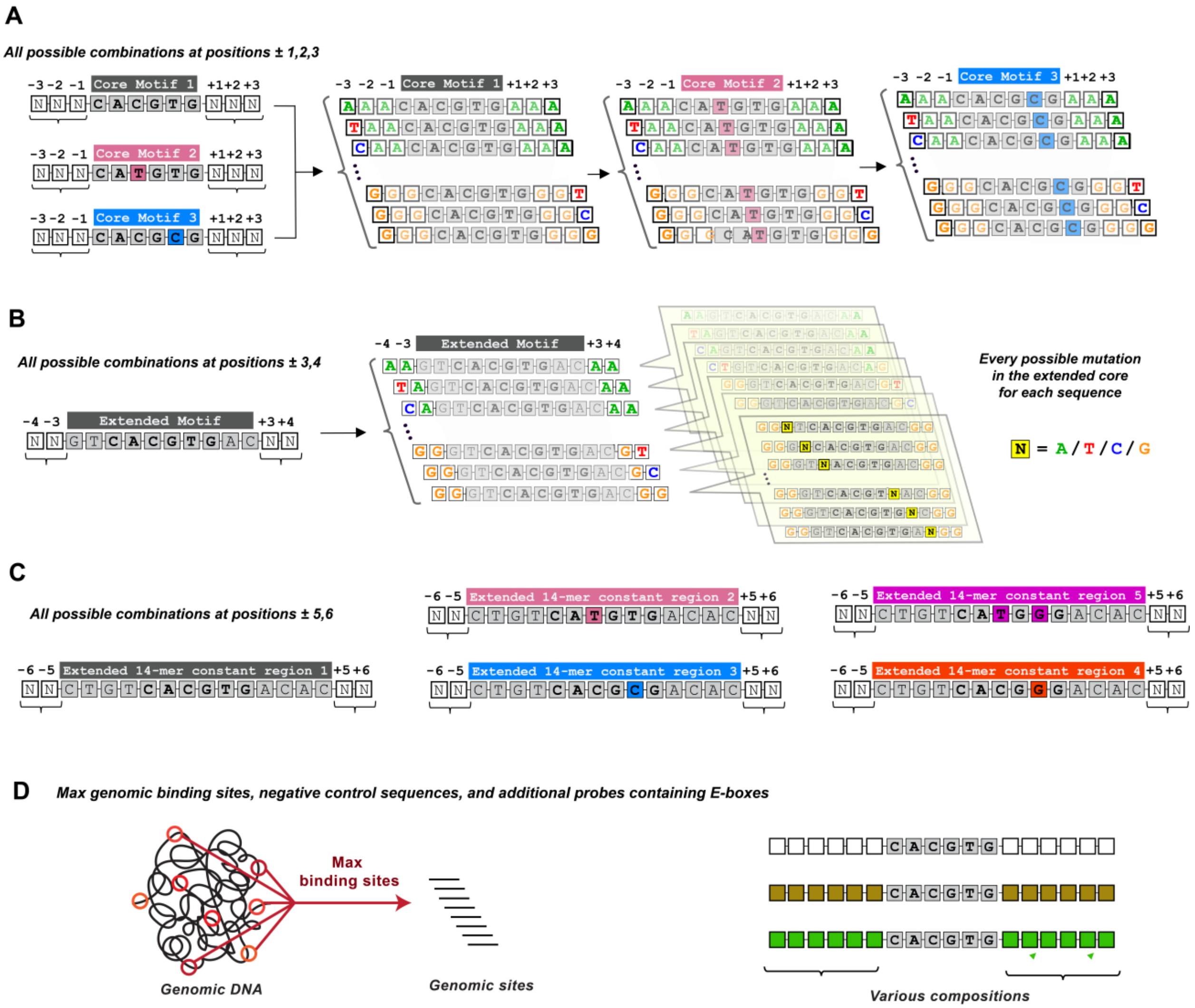


**Figure S23.** Schematic representation of libraries designed to explore binding differences beyond the core 8-mer motif. **(A)** We selected three 6-bp-long cores and varied the three neighboring bases on each side to every possible combination, resulting in 4,096 combinations for each of the three cores. **(B)** To further explore the bases 3 and 4 bases away from the E-box, we chose an extended 10-bp core, and varied positions ±3,4 to all possible options. For each of these options, we varied each possible base in the extended core region. **(C)** To explore the distal positions 5 and 6, we selected five different 14-mer constant regions, and varied positions +5,6 to all possible combinations. **(D)** In addition to these sequences, we also included genomic sequences previously shown to be bound by Max,[10,11] as well as additional sites not expected to bind Max as a negative control, and additional sequences containing the core motif in various flanking environments.

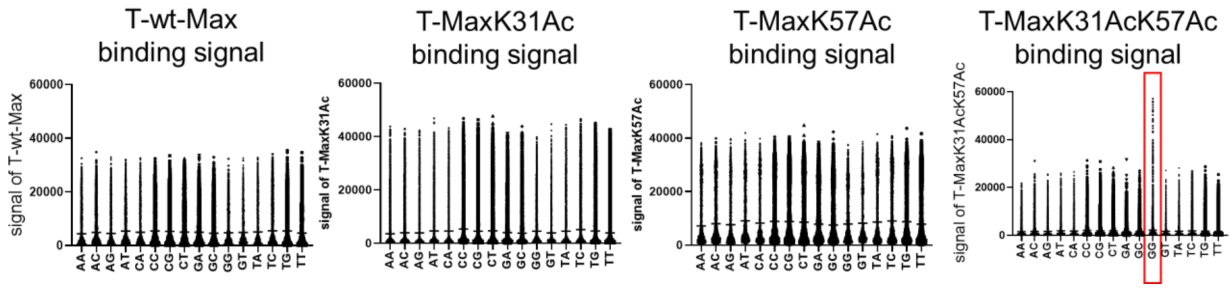


**Figure S24.** The signal distribution of **T-wt-Max** and **T-MaxK31AcK57Ac** for all possible nucleotide combinations at positions +3 and +4 is depicted. In the case of **T-MaxK31AcK57Ac** (far right), a subgroup with the GG combination (highlighted by a red rectangle) exhibits significantly higher binding signals compared to all other combinations (t-test p-value<0.0001). This observation highlights a distinct preference of **T-MaxK31AcK57Ac** for certain sequences with GG at these positions.

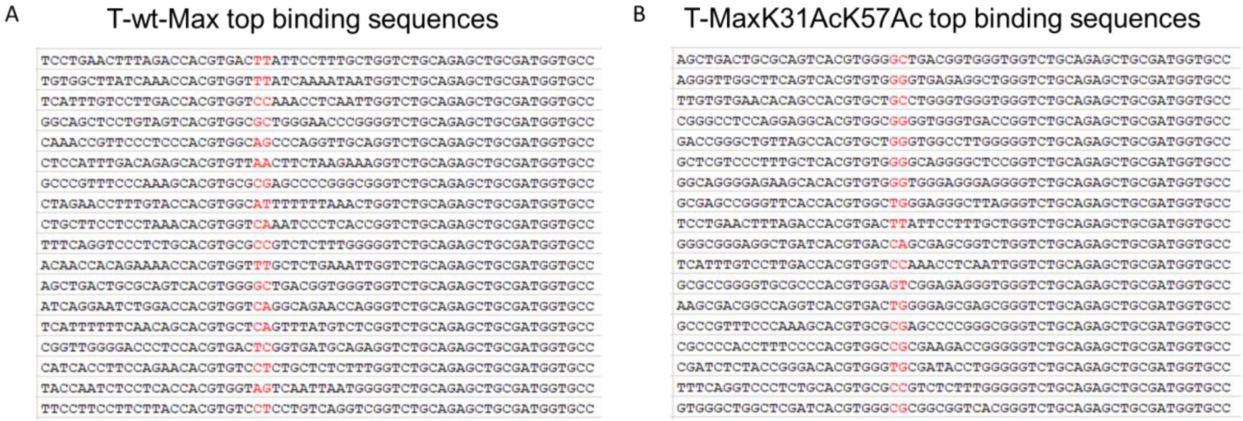


**Figure S25.** Comparison of the top 5% strongest binders within the genomic binding sites group for (**A**) **T-wt-Max** and (**B**) **T-MaxK31AcK57Ac**. The positions +3 and +4 are highlighted in red. Notably, while no GG motif is present in these positions for WT-Max (A), the top binders of **MaxK31AcK57Ac** (B) are enriched with GG, constituting approximately 27% of the sequences.

# 12. References


1. Zheng, J.-S., Tang, S., Qi, Y.-K., Wang, Z.-P. & Liu, L. Chemical synthesis of proteins using peptide hydrazides as thioester surrogates. *Nat Protoc* **8**, 2483–2495 (2013).

2. Dawson, P. E., Muir, T. W., Clark-Lewis, I. & Kent, S. B. H. Synthesis of Proteins by Native Chemical Ligation. *Science (1979)* **266**, 776–779 (1994).

3. Fang, G. *et al.* Protein Chemical Synthesis by Ligation of Peptide Hydrazides. *Angewandte Chemie International Edition* **50**, 7645–7649 (2011).

4. Wan, Q. & Danishefsky, S. J. Free-Radical-Based, Specific Desulfurization of Cysteine: A Powerful Advance in the Synthesis of Polypeptides and Glycopolypeptides. *Angewandte Chemie International Edition* **46**, 9248–9252 (2007).

5. Haase, C., Rohde, H. & Seitz, O. Native Chemical Ligation at Valine. *Angewandte Chemie International Edition* **47**, 6807–6810 (2008).

6. Nithun, R. V. *et al.* Deciphering the Role of the Ser-Phosphorylation Pattern on the DNA-Binding Activity of Max Transcription Factor Using Chemical Protein Synthesis. *Angewandte Chemie* **135**, (2023).

7. Berger, M. F. & Bulyk, M. L. Universal protein-binding microarrays for the comprehensive characterization of the DNA-binding specificities of transcription factors. *Nat Protoc* **4**, 393–411 (2009).

8. Berger, M. F. *et al.* Compact, universal DNA microarrays to comprehensively determine transcription-factor binding site specificities. *Nat Biotechnol* **24**, 1429–1435 (2006).

9. Mukherjee, S. *et al.* Rapid analysis of the DNA-binding specificities of transcription factors with DNA microarrays. *Nat Genet* **36**, 1331–1339 (2004).

10. Afek, A., Schipper, J. L., Horton, J., Gordân, R. & Lukatsky, D. B. Protein−DNA binding in the absence of specific base-pair recognition. *Proceedings of the National Academy of Sciences* **111**, 17140–17145 (2014).

11. Zhou, T. *et al.* Quantitative modeling of transcription factor binding specificities using DNA shape. *Proceedings of the National Academy of Sciences* **112**, 4654–4659 (2015).
